# Depicting Primate-Like Granular Dorsolateral Prefrontal Cortex in the Chinese Tree Shrew

**DOI:** 10.1523/ENEURO.0307-24.2024

**Published:** 2024-10-24

**Authors:** Xiu-Peng Nie, Xiao-Shan Xu, Zhao Feng, Wei Wang, Chen Ma, Yue-Xiong Yang, Jin-Nan Li, Qi-Xin Zhou, Fu-Qiang Xu, Min-Hua Luo, Jiang-Ning Zhou, Hui Gong, Lin Xu

**Affiliations:** ^1^University of Chinese Academy of Sciences, Beijing 100049, China; ^2^Chinese Academy of Science Key Laboratory of Animal Models and Human Disease Mechanisms, and KIZ-SU Joint Laboratory of Animal Model and Drug Development, and Laboratory of Learning and Memory, Kunming Institute of Zoology, Kunming 650223, China; ^3^Britton Chance Center for Biomedical Photonics, Wuhan National Laboratory for Optoelectronics-Huazhong University of Science and Technology, Wuhan 430074, China; ^4^HUST-Suzhou Institute for Brainsmatics, JITRI, Suzhou 215123, China; ^5^Shen Zhen Institute of Advanced Technology, Chinese Academy of Sciences, Xi Li Shen Zhen University Town, Shenzhen 518055, China; ^6^Chinese Academy of Sciences Centre for Excellence in Brain Science and Intelligent Technology, Shanghai 200031, China; ^7^State Key Laboratory of Virology and Wuhan Institute of Virology, Chinese Academy of Sciences, Wuhan 430071, China; ^8^Institute of Brain Science, the First Affiliated Hospital of Anhui Medical University, Hefei 230022, China; ^9^Chinese Academy of Science Key Laboratory of Brain Function and Diseases, School of Life Sciences, University of Science and Technology of China, Hefei 230027, China

**Keywords:** dorsolateral prefrontal cortex (dlPFC), fourth layer (L4), granular cell, medial prefrontal cortex (mPFC), mediodorsal nucleus (MD), orbital frontal cortex (OFC), primate, tree shrew

## Abstract

It remains unknown whether the Chinese tree shrew, regarded as the closest sister of primate, has evolved a dorsolateral prefrontal cortex (dlPFC) comparable with primates that is characterized by a fourth layer (L4) enriched with granular cells and reciprocal connections with the mediodorsal nucleus (MD). Here, we reported that following AAV-hSyn-EGFP expression in the MD neurons, the fluorescence micro-optical sectioning tomography revealed their projection trajectories and targeted brain areas, such as the hippocampus, the corpus striatum, and the dlPFC. Cre-dependent transsynaptic viral tracing identified the MD projection terminals that targeted the L4 of the dlPFC, in which the presence of granular cells was confirmed via cytoarchitectural studies by using the Nissl, Golgi, and vGlut2 stainings. Additionally, the L5/6 of the dlPFC projected back to the MD. These results suggest that the tree shrew has evolved a primate-like dlPFC which can serve as an alternative for studying cognition-related functions of the dlPFC.

## Significance Statement

This study depicts the coordinates of the dorsolateral prefrontal cortex (dlPFC) in tree shrews, which have remained unknown previously, by using the standard classic methods and the fluorescence micro-optical sectioning tomography. As the tree shrew is known to be the living closest sister of primates in genome, this study would be important for the research fields of dlPFC structure and function and suggests that the tree shrew can serve as an alternative species for studying the granular dlPFC-related cognitive functions.

## Introduction

The family Tupaiidae, known also as tree shrews, is an order still not fully resolved in terms of taxonomy. It is classified as its own order, Scandentia (Peng et al., 1991; Zheng et al., 2014), or as a suborder of Primates (Buettner-Janusch, 1963; Martin, 1968; Clark, 1971). However, an analysis of the genome of the Chinese tree shrew (*Tupaia belangeri chinensis*), which is distributed in Southwest China such as the Yunnan Province (Peng et al., 1991; Zheng et al., 2014), suggests that this species may be the closest living relative of primates (Y. Fan et al., 2013).

The Chinese tree shrew has been domesticated in the Primates Center of Kunming Institute of Zoology for over 50 years (Peng et al., 1991; Wang et al., 2013b; Zheng et al., 2014), making it a promising animal for neuroscience research (Savier et al., 2021) or drug safety testing (Cao et al., 2003). Its body mass ∼150 g and fast breeding of three to five babies per gestation period for 45–50 d (Peng et al., 1991; Wang et al., 2013b; Zheng et al., 2014) give it a high cost-effectiveness. Studies on tree shrews have led to important findings (Xu et al., 2013), such as those under social conflict (Fuchs et al., 1995; Gould et al., 1997) well supporting the stress etiology hypothesis of major depressive disorder (MDD; Fuchs et al., 1996; Wang et al., 2012, 2013a; Shu et al., 2015; L. Fang et al., 2016). Tree shrews also have a sleep structure similar to that of humans (Dimanico et al., 2021), making them an ideal model for sleep research (Berger and Walker, 1972; S. Y. Liu et al., 1982). Furthermore, tree shrews have a highly similar visual system to that of primates (Fitzpatrick, 1996; Zhang et al., 2018; Savier et al., 2021). Stereotaxic atlas (J. N. Zhou and Ni, 2016; Ni et al., 2018a) and magnetic resonance imaging and positron emission tomography templates (Huang et al., 2018) for the tree shrew brain have been developed.

The relationship between tree shrews and primates in aspects of brain structure and function remains still unclear. Studies have suggested that the prefrontal cortex (PFC) may be the highest level of the cortical hierarchy playing a critical role in higher cognitive functions in primates (Goldman-Rakic, 1990; Fuster, 2001; Carlen, 2017). According to Brodmann's cytoarchitecture definition of the PFC (1909), it is the frontal cortex (FC) subregions characterized by a granular layer IV (L4), which is widely accepted as a specific feature of primates (Brodmann, 1909; Carlen, 2017). However, this definition may not be appropriate for other mammals such as the widely used murine (mouse or rat) or the some nonsimian primates lacking the evidence of the dorsolateral PFC (dlPFC; Preuss and Wise, 2022). Fortunately, Rose and Woolsey (1948) proposed that the PFC is a part of the FC receiving the mediodorsal nucleus (MD) projection. Accordingly, Divac et al. (1978) used horseradish peroxidase tracing to find the PFC of the tree shrew (*T. belangeri*) but the authors did not measure either stereotaxic coordinates or cytoarchitecture of the structure (Divac et al., 1978). Later studies in murine showed that the MD only projected to the medial and orbital FC (OFC) but not the dorsolateral part (Leonard, 1969; 2016; Guldin et al., 1981), leading to the conclusion that the medial region of the FC is the mPFC, which is regarded as potentially a homologous region of the mPFC in primates. However, the MD has been shown to project to all subregions of the PFC in primates (Wise, 2008; Carlen, 2017). Recent studies on the PFC have all been consistent in their interpretation (Carlen, 2017) that the PFC in primates can be characterized by an L4 with either granular (conspicuous L4 with dense granular cells) in the anterior parts or dysgranular (thinner L4 with lesser granular cells) in the posterior parts (Wise, 2008; L. Fan et al., 2016; Carlen, 2017). This is in sharp contrast to the fact that all of the FC subregions including the mPFC in murine are agranular (lacking the L4 and granular cells; Lein et al., 2007).

In the present study, rigorous criteria are used to define the PFC in the Chinese tree shrew. Classic methods as well as newly developed techniques such as fluorescence micro-optical sectioning tomography (fMOST; Gong et al., 2013, 2016; Zhong et al., 2021) are used to identify the dlPFC of the tree shrew. We find that the dlPFC is characterized by an L4 enriched with granular cells that receive MD projections and that the L5/6 of the dlPFC sends projections back to the MD. This indicates that the tree shrew has evolved a primate-like dlPFC, which is likely related to higher cognitive functions.

## Materials and Methods

### Animals

Twenty-two adult male tree shrews (*T. belangeri chinensis*) over 1.5 years old (the lifespan of a tree shrew is ∼6 years in the Primate Center) and ∼130 g were used in this study. The tree shrews were bred and housed at the Primates Center of Kunming Institute of Zoology, the Chinese Academy of Sciences, Kunming 650223, Yunnan, China. The tree shrews were housed individually in a thermoregulated room (26 ± 2°C) with food and water *ad libitum*. Relative humidity was appropriately set between 40 and 70%. The noise was kept below 60 dB, and the illumination was set automatically from 8:00 A.M. to 8:00 P.M. All animal care and experimental protocols were approved and supervised (SMKX-20190106-02) by the Animal Ethics Committee of Kunming Institute of Zoology, the Chinese Academy of Sciences, Kunming 650223, Yunnan, China.

### Viral injection surgery

The experimental procedures were like those described previously (Wang et al., 2013a,b; Ni et al., 2020). Briefly, the tree shrew was anesthetized by intraperitoneal injection of pentobarbital sodium (80 mg·kg^−1^, from Sigma-Aldrich). After anesthesia, the body was placed on a surgical heating pad. The head was mounted on a stereotaxic apparatus and supplied oxygen through a nose-clip mask (RWD Life Science). The tree shrew's hair was cut off and the scalp was cleaned with alcohol. Then, the scalp was opened by using surgical blade, and the subcutaneous tissue and blood were removed and then washed with sterile saline. The skull surface was cleaned and the sagittal suture was exposed. The anterior and posterior fontanelle was adjusted at the same level according to the Tree Shrew (*Tupaia belangeri chinensis*) Brain in Stereotaxic Coordinates (J. N. Zhou and Ni, 2016). The skull at the injection site was drilled with a cranial drill without piercing the dura, and the virus (100–200 nl) was injected into the targeted brain region at a rate of 30 nl·min^−1^ by using the glass micropipettes (WPI, World Precision Instruments) connected to a microsyringe pump (Nanoliter 2010, WPI). After the viral injection, the glass micropipettes stayed still for 10 min to allow the injected virus to be fully diffused. The scalp was sutured and disinfected with medical hydrogen peroxide. The tree shrew was not placed back into his home cage until awaking from the anesthesia, and a certain amount of antibiotics was added to the food for avoiding infection and anti-inflammation. In addition, the tree shrew was fed by mealworms to help his recovery from the surgery. After viral expression, the tree shrew was killed by overdose pentobarbital sodium anesthesia and perfused transcranial with the phosphate-buffered saline (PBS), pH 7.4, and followed by 4% paraformaldehyde (PFA). The tree shrew brain was removed and immersed in PBS containing 30% sucrose. The brain was then sectioned by using a freezing microtome (RWD Life Science) at 50 μm thickness and stained with 0.1% DAPI and then imaged by using Olympus confocal imaging system FV3000 (Olympus).

### Viruses, stereotaxic coordinates, and viral tracing

**Figure 1.**
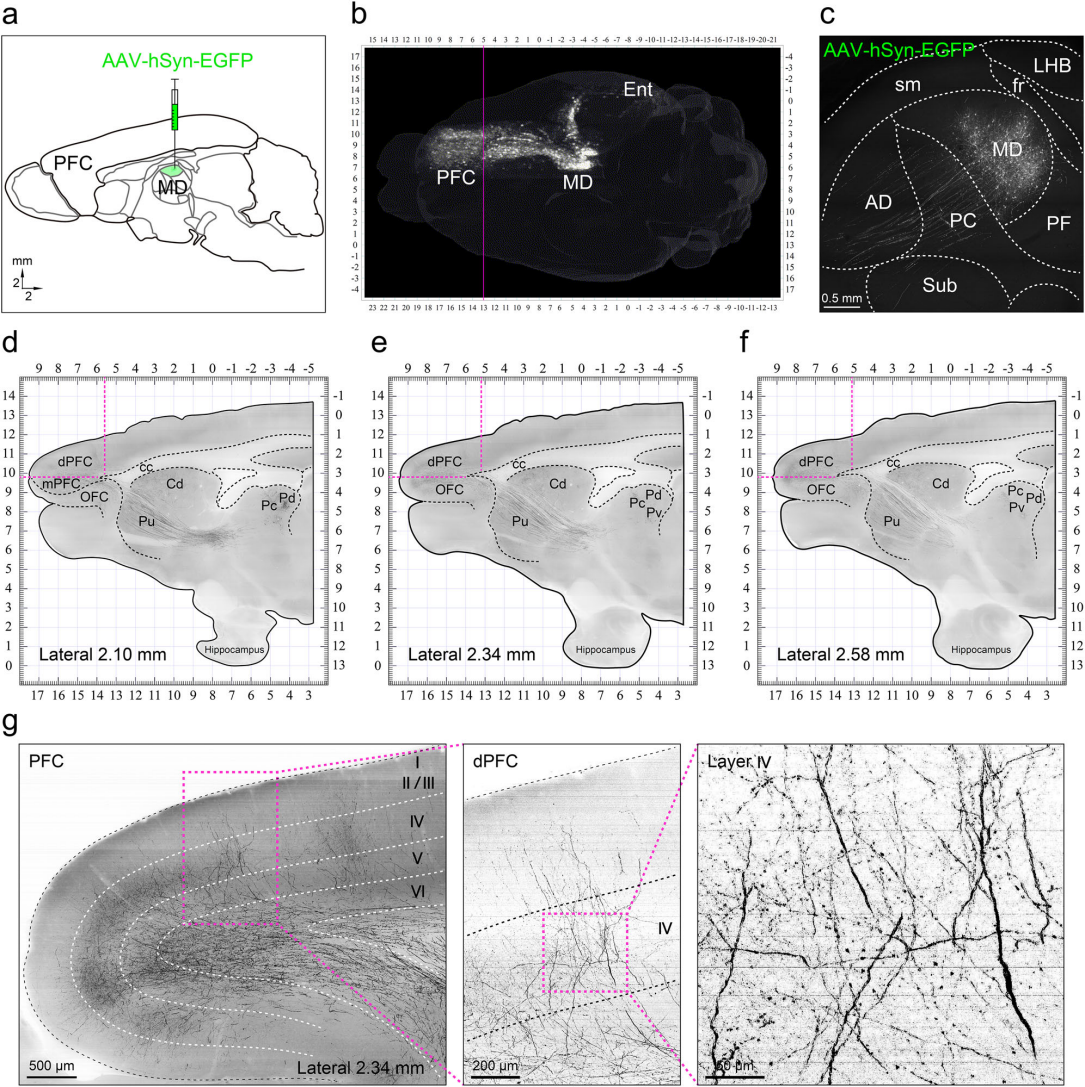
The fMOST delineates the MD→PFC in the Chinese tree shrew. ***a***, Injection of AAV-hSyn-EGFP into the mediodorsal nucleus (MD). ***b***, ***c*** The fMOST clearly visualized the EGFP-labeled neurons within the MD and the MD projection trajectories and targeting brain regions. ***d–f***, The MD projection to the caudate nucleus (Cd), the putamen (Pu), and densely to the dorsal, medial, and orbital subregions of the frontal cortex (FC). ***g***, The MD projection terminals located in the dorsal frontal cortex (dPFC), forming presynaptic boutons in the cortical layers of the dPFC. fMOST, fluorescence micro-optical sectioning tomography; PFC, prefrontal cortex; Ent, entorhinal cortex. AD, anterodorsal nucleus; PC, paracentral nucleus; sm, stria medullaris; Sub, submedius nucleus; fr, fasciculus retroflexus; PF, parafascicular nucleus; LHB, lateral habenula. mPFC, medial PFC; OFC, orbital FC; cc, corpus callosum; Pc, central nucleus of the pulvinar; Pd, dorsal nucleus of the pulvinar; Pv, ventral nucleus of the pulvinar.

**Figure 2.**
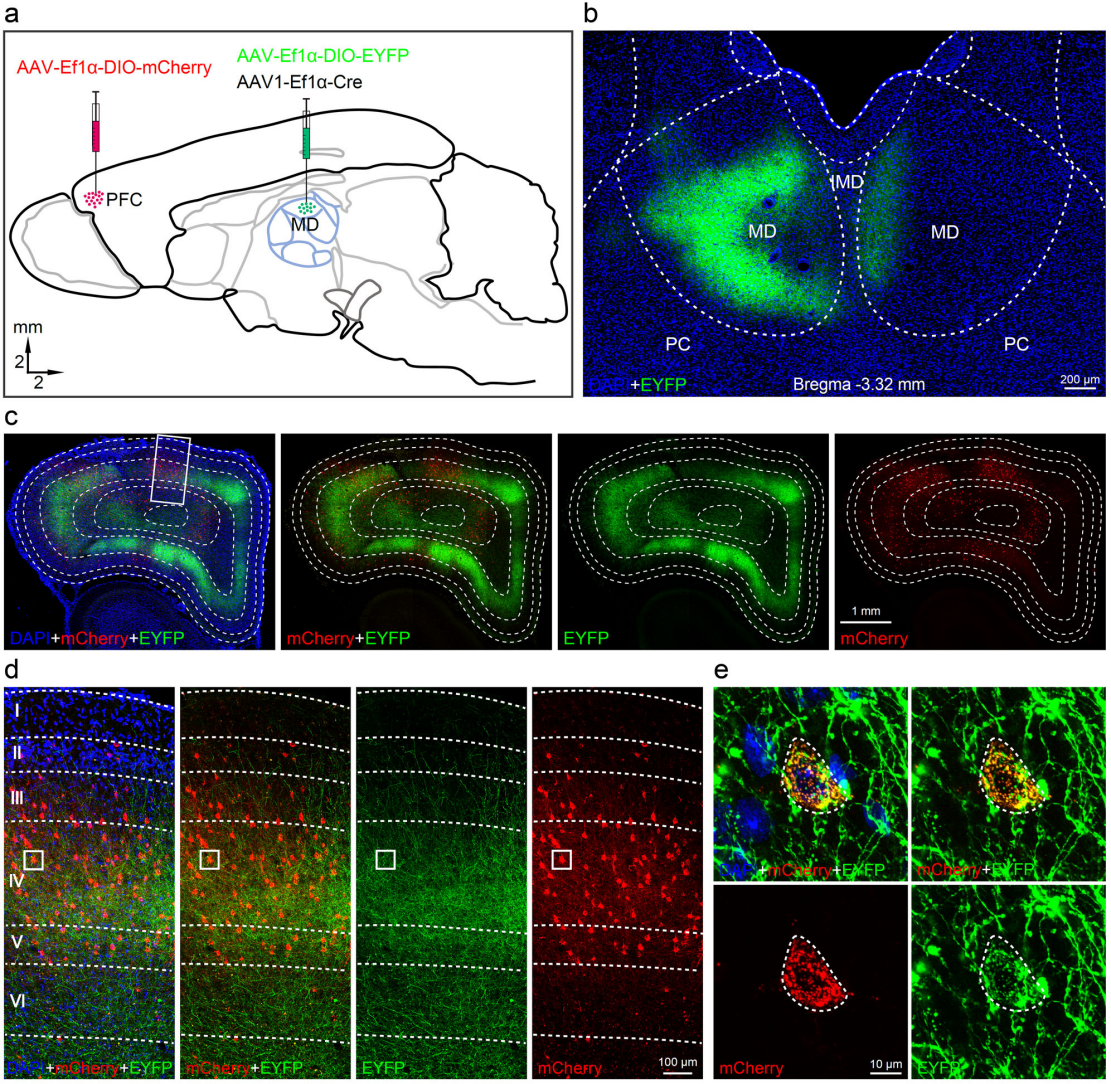
Cre-dependent tracing indicates the MD-projection to the L4 of the dPFC in tree shrews. ***a***, AAV-Ef1α-DIO-EYFP and AAV1- Ef1α-Cre were injected into the MD, but AAV- Ef1α-DIO-mCherry alone was injected into the dPFC. ***b***, The EYFP-labeled neurons were observed within the MD of the viral injecting side and the EYFP-labeled terminals were found in the contralateral MD. ***c***, The mCherry-labeled neurons in the dPFC were resulted from anterograde transferring of the Cre from the MD that met the expression of AAV-Ef1α-DIO-mCherry. ***d***, ***e***, These mCherry-labeled neurons were mainly located in the L4 of the dPFC where the MD-projected terminals were densely distributed. MD, mediodorsal nucleus; IMD, intermediodorsal nucleus; PC, paracentral nucleus; dPFC, dorsal prefrontal cortex.

The viral tracing methods used here were like those described previously (H. Zhou et al., 2017; W. Zhou et al., 2019). The viruses used here were all purchased from BrainVTA and listed as the following: (1) AAV-hSyn-EGFP or mCherry (AAV2/9-hSyn-EGFP/mCherry-WPRE-pA) was injected into the MD at the stereotaxic coordinates: (−3.32, 0.8, −5.5) [anterior–posterior (AP), medial–lateral (ML), dorsal–ventral (DV) in mm] or the dlPFC at (+7.23, 3, −3.0) or the mPFC (+6.7, 0.6, −4.3) or (+5.16, 0.6, −4.0) for visualizing the projection trajectories and targeted brain regions. The non-transsynaptic retrograde tracing rabies virus was injected into the ventral subiculum at the coordinates (−3.32, 2.0, −13.4) (Fig. 1; Extended Data Fig. 1-1, 1-2, 1-3, 3-1). (2) AAV-Ef1α-DIO-EYFP (AAV2/9-Ef1α-DIO-EYFP-WPRE-pA) and anterograde transsynaptic virus AAV1-Ef1α-Cre (AAV1-Ef1α-Cre-WPRE-pA) were injected into the MD (−3.32, 0.8, −5.4); meanwhile AAV-Ef1α-DIO-mCherry (AAV2/9-Ef1α-DIO-mCherry-WPRE-pA) was injected into the dlPFC (+7.0, 3.0, −2.8) (Fig. 2). (3) AAVretro-hSyn-GFP-Cre-WPRE-pA was injected into the dlPFC (+7.1, 3.0, −2.9) and AAV2/9-Ef1α-DIO-mCherry-WPRE-pA was injected into the MD/PC (−3.32, 0.8, −5.6). AAV2/9-hSyn-FLExFRT-Mgfp-2A-Synaptophysin-mRuby-WPRE-pA and AAV2/9-EF1α-FLP-WPRE-pA helper virus were both injected into the dlPFC (+7.0, 3.0, −2.8) (Extended Data Fig. 2-1). (4) The non-transsynaptic retrograde tracing rabies viruses RV-ΔG-N2C-EGFP or DsRed were used to trace the projection neurons in either the thalamus (the MD, the PC, and the IMD) or the PFC (the dlPFC and the mPFC). The injection stereotaxic coordinates: the MD (−3.19, 0.8, −5.4), the PC (−3.32, 1.4, −6.5), the IMD (−3.45, 0, −6.0), the dlPFC (+7.2, 3.0, −3.0), and the mPFC (+ 7.5, 0.6, −4.4) or (+6.5, 0.6, −4.2) or (+5.2, 0.6, −4.0) (Figs. 5, 6; Extended Data Fig. 2-2).

10.1523/ENEURO.0307-24.2024.f1-1 Figure 1-1 **The MD neurons project to the entorhinal cortex (Ent) and the cornu ammonis (CA) areas of the hippocampal formation, and to the primates-like corpus striatum.** (a) The EGFP-labelled neurons within the MD project to the Ent and the lacunosum molecular layer (LMoL) of the CA areas. (b) The EGFP-labelled neurons within the MD also project to the corpus striatum, which developed to be a primates-like structure. VS = ventral subiculum; DS = dorsal subiculum; MoDG = molecular layer of the dentate gyrus (DG). Cd = caudate nucleus; Pu = putamen; ic = internal capsule; acp = anterior commissure posterior part; STL = bed nucleus of the stria terminalis lateral division; st = stria terminalis; aci = anterior commissure intrabulbar part; Acb = accumbens nucleus; EGP = external globus pallidus. Download Figure 1-1, TIF file.

10.1523/ENEURO.0307-24.2024.f1-2 Figure 1-2 **The stereotaxic coordinates for the dPFC, mPFC, and OFC**. According to the Tree Shrew (*Tupaia belangeri chinensis*) Brain in Stereotaxic Coordinates (Zhou & Ni, 2016) and the present fMOST data, we delineated the dPFC, mPFC, and OFC in the Chinese tree shrew. dPFC = dorsal prefrontal cortex; mPFC = medial prefrontal cortex; OFC = orbital frontal cortex; cc = corpus callosum; Cl = clastrum; fmi = forceps minor of the corpus callosum. Download Figure 1-2, TIF file.

**Figure 3.**
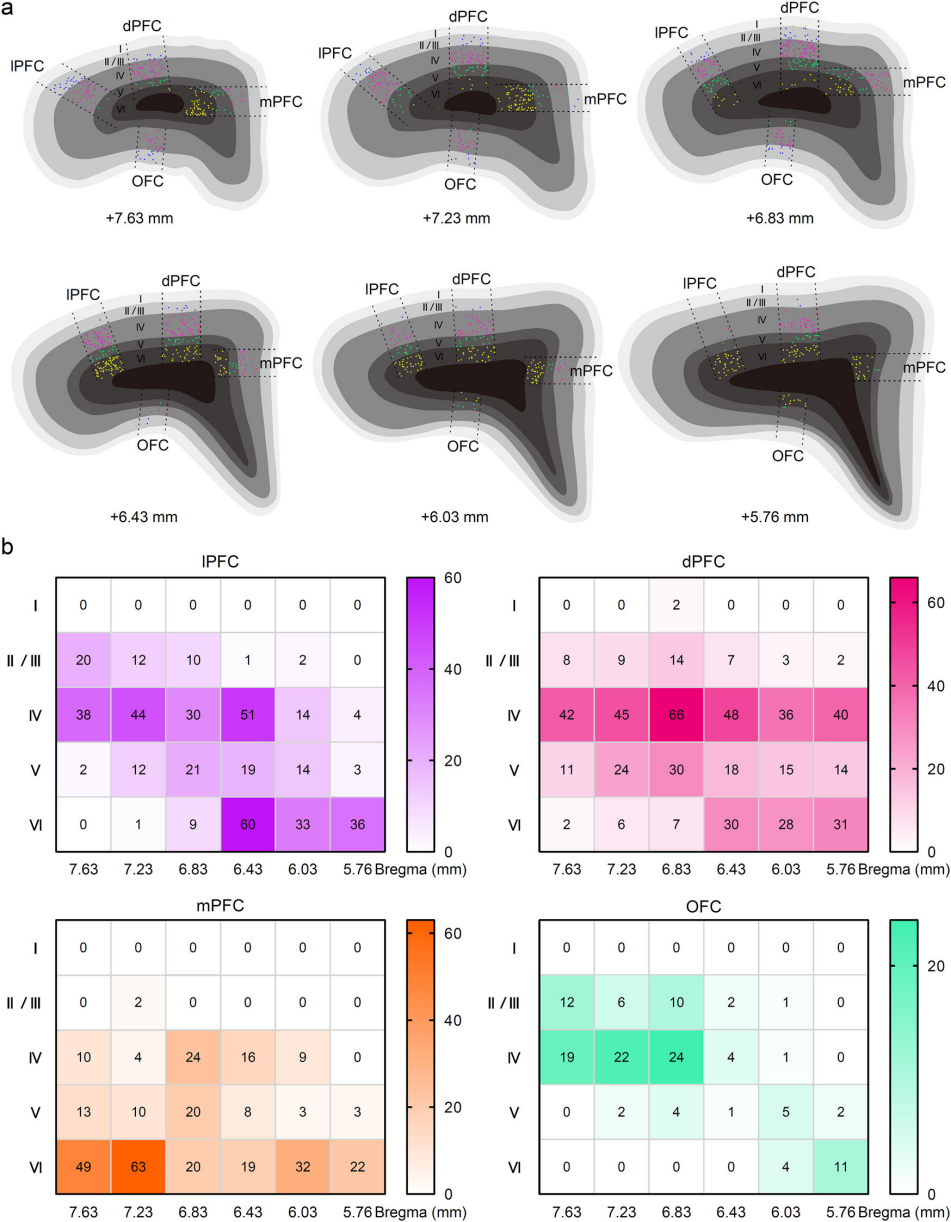
Definition of the dorsolateral prefrontal cortex (dlPFC) in the tree shrew. ***a***, The colors indicated layer 2/3 (blue), layer 4 (red), layer 5 (green), and layer 6 (yellow) in the PFC. Each dot represents a neuron that received Cre from the MD to drive mCherry expression. Each panel indicated the anterior–posterior coordinates (AP) from the bregma: +7.63, +7.23, +6.83, +6.43, +6.03, and +5.76 mm. ***b***, The distribution of neuronal numbers in the layers that received the MD projections in the lateral PFC (lPFC), dorsal PFC (dPFC), medial PFC (mPFC), and orbital FC (OFC) at the AP coordinates. Notably, the pattern of the lPFC and dPFC was highly similar and pooled together as the dlPFC in the tree shrew.

10.1523/ENEURO.0307-24.2024.f1-3 Figure 1-3 **Non-transsynaptic retrograde tracing virus RV-ΔG-N2C-EGFP confirms direct projections from the mediodorsal nucleus (MD) to the ventral subiculum (VS) of the hippocampus.** (a) RV-ΔG-N2C-EGFP was injected into the ventral subiculum (VS). (1) The injection site in the VS. (2) Neurons were labeled within the MD with (left) or without DAPI (right) signal, suggesting that the MD projects directly to the VS. (3-4) The neurons in the L2/3 of the entorhinal cortex (Ent) were also labeled with (3) or without DAPI (4) signal, suggesting that the Ent L2/3 directly projects to the Vs. Rad = radiatum layer of the CA areas. D3  V = third ventricle; PVP = paraventricular nucleus posterior part; IMD = intermediodorsal nucleus. Download Figure 1-3, TIF file.

10.1523/ENEURO.0307-24.2024.f2-1 Figure 2-1 **Reciprocal connections between the dlPFC and the MD.** (a) AAVretro-hSyn-GFP-Cre-wpre-pA was injected into the dlPFC and AAV-Ef1α-DIO-mCherry-wpre-pA was injected into the MD. (b) The GFP-labelled neurons were observed in the 4-6 layers of the dlPFC, where the expressed Cre can be retrogradely transferred to the projection neurons of the MD. (c) The retrograde Cre then drove mCherry expression (AAV-Ef1α-DIO-mCherry-wpre-pA) in the MD and paracentral nucleus (PC), indicating that these mCherry-labelled neurons projected to the GFP-labelled neurons in the dlPFC. Conversely, the GFP-labelled neurons sent projection terminals to surround the mCherry-labelled neurons in the MD. (d) AAV-hsyn-FLExFRT-Mgfp-2A-Synaptophysin-mRuby-WPRE-pA and AAV-EF1α-FLP-WPRE-Pa helper were injected into the dlPFC. (e) Both Mgfp (Green) and mRuby (Red) labeled neurons were observed in the 4-6 layers of the dlPFC. (f) The dlPFC-projection terminals showed denser mRuby than Mgfp signals in the MD and PC. IMD = intermediodorsal nucleus; CM = central medial nucleus; Sub = submedius nucleus. Download Figure 2-1, TIF file.

10.1523/ENEURO.0307-24.2024.f2-2 Figure 2-2 **Neuronal numbers project to the MD, PC, and IMD.** (a-c) The topographical distribution of neuronal numbers in the dlPFC, mPFC, and OFC projected to the MD, PC, and IMD. The colors represented neurons (each dot = a neuron) in the dlPFC (yellow), mPFC (red), and OFC (green). (d) From the AP +6.25 to +4.09  mm indicating neuronal numbers projected to the MD, which were increasing in the mPFC while decreasing in the dlPFC and OFC. (e) Following the AP from +6.25 to +4.09  mm for neurons projecting to the PC, neuronal numbers of the mPFC, dlPFC, and OFC remained unchanged. (f) As the AP at +7.1 to +6.03  mm, neuronal numbers projecting to the IMD were decreasing. Download Figure 2-2, TIF file.

10.1523/ENEURO.0307-24.2024.f3-1 Figure 3-1 **Comparison of the dlPFC and mPFC in descending projections to the thalamic nuclei.** (a) AAV-hsyn-EGFP-WPRE-pA or AAV-hsyn-mCherry-WPRE-pA was injected into the dlPFC or mPFC, respectively. (b) The MD, IMD, and PC were the common regions receiving both the dlPFC and mPFC projections. (c-d) The EGFP-labelled neurons covered the L2-6 of the mPFC, in which the L4 looks like dsygranular. These neurons projected to the MD, PC, and IMD, with very few in the contralateral side of the MD. (e-f) The mCherry-labelled neurons were distributed also in the L2-6 of the mPFC, in which the L4 was much denser at this coordinate. These neurons sent projection terminals to the PC mainly. (g-h) The mCherry-labelled neurons were also distributed in the L2-6 of the dlPFC, where the L4 enriched with granular cells. These neurons projected to the MD, PC, IMD, CM, and Sub. PrL = prelimbic cortex; IMD = intermediodorsal nucleus; PC = paracentral nucleus; CM = central medial nucleus; Sub = submedius nucleus. Download Figure 3-1, TIF file.

The AAV virus titer was ∼10^13^ vg·ml^−1^. The RV virus titer was ∼10^8^ vg·ml^−1^. The period for AAV expression after injection was 21–28 d; that for RV expression after injection was 5–7 d.

### Immunofluorescence staining

The adult male tree shrews were killed by using overdose pentobarbital sodium anesthesia and perfused intracardially with PBS, pH 7.4, followed by 4% PFA. The brain was postfixed in 4% PFA overnight and dehydrated in 15% sucrose in PBS and then maintained in 30% sucrose in PBS at 4°C. The brain was then sectioned by using a freezing microtome (RWD Life Science) at 50 μm thickness of coronal sections. The brain sections were kept at −20°C with antifreeze buffer (20% glycerol, 30% ethylene glycol, and 50% PBS). The brain slices were removed from antifreeze buffer and put in 1× PBS for 30 min at room temperature. The slices were washed three times in PBS 10 min each time and then soaked in cell membrane lysate (0.6% Triton X-100 in PBS, Sigma-Aldrich) for 30 min. The slices were put in sodium citrate solution at 95°C for 4 min for metal bath antigen retrieval and then washed three times with PBS, 10 min each time after cooling. The slices were blocked in 5% bovine serum albumin (BSA, RD Bioscience) for 1 h, and then the primary antibodies (anti-TBR1 1: 400, anti-CB 1:300, anti-PV 1:300, and anti-VGLU2 1:300 in 1% BSA) were added and placed in the incubation plate on a shaker for 4°C overnight. The slices were placed at room temperature for 30 min and washed three times with PBS, 10 min each time, then secondary antibody was added, and the slices were incubated in the dark for 1.5 h. After that, the slices were washed three times with PBS, 10 min each time, and placed on the slide and dried away avoiding light, then the sealing agent (solvent: 70% glycerol, 30% 1× PBS; DAPI 1:1,000, Thermo Fisher Scientific) was added, and the slide was covered. Anti-T-box brain protein 1 antibody (TBR1, Rabbit), Anti-Parvalbumin antibody (PV, Rabbit), Anti-Vesicular glutamate transporter 2 antibody (VGLU2, Rabbit), Anti-Calbindin antibody (CB, Mouse), Goat Anti-Rabbit IgG H&L (Alexa Fluor 594), Donkey Anti-Rabbit IgG H&L (Alexa Fluor 488), and Goat Anti-Mouse IgG H&L (Alexa Fluor 488) were purchased from Abcam.

### The Nissl staining

The procedures for the brain sections were like those in the above. Then, the brain slices were placed onto slides and dried in the shade. The slices were placed in 75% ethanol for 4 h, followed by 0.1% tar violet staining solution (Solarbio Science & Technology) for 4 h. The slices were washed twice with distilled water, then 70% ethanol solution for 5 min, 95% ethanol solution for 3 min, and anhydrous ethanol for 10–20 s for dehydration. The slices were soaked with xylene twice for transparent tissue 10 min each time and then sealed with neutral resin. The basophilic substances in neuronal bodies or dendrites could be colored by the tar violet solution. Neurons can be classified according to the size, shape, number, and color depth of the Nissl bodies.

### The Golgi staining

Adult male tree shrews were killed by using overdose pentobarbital sodium anesthesia. The brain was quickly removed and washed with distilled water. FD Rapid Golgistain Kit (FD NeuroTechnologies) was used for the staining. The fresh brain tissue was stored at room temperature for 2 weeks in a day-ahead prepared A + B mixed solution. The A + B mixed solution was replaced every day and was unstirred to avoid precipitation. The brain tissue was then immersed in solution C for 3 d and stored in the dark at room temperature. Solution C was replaced every day. After that, the brain tissue was sectioned at 120 μm thickness by using a vibrating slicer and washed twice with double distilled water, 10 min each time. The slices were immersed in the mixture of solution D + solution E + distilled water (volume ratio, 1:1:2) for 15 min and then rinsed with double distilled water twice, 10 min each time. The slices were dehydrated in a gradient of 50%, 75%, and 95% ethanol, 10 min each gradient. The slices were made transparent by using xylene three times, 10 min each, and sealed with neutral resin.

### The fMOST

The procedures of the fMOST are like those described previously (A. Li et al., 2010; Gong et al., 2013; Sun et al., 2019). Briefly, the tree shrew brain was embedded with agarose before data acquisition. The oxidized agarose with a concentration of 5% was made by adding 10 mM sodium periodate at room temperature. Then the fixed brain was immersed into a homemade container filled with the oxidized agarose in the 55°C water bath for 0.5 h. At last, the embedded brain with the container was left at room temperature for 0.5 h until the complete solidification of the agarose. After that, the embedded brain was imaged by using the previously developed fMOST system automatically (A. Li et al., 2010; Gong et al., 2013; Sun et al., 2019). On the fMOST system, the agarose embedded brain was fixed in a water tank, immersing with PBS. The surface of the brain with a thickness of 8 μm was removed by using the homemade large-scale vibratome. Then four images with a depth interval of 2 μm were acquired by using line-scanning microscopic imaging with 20× objective. To alleviate the image degeneration induced by the rough surface after mechanical sectioning, the initial image was acquired at the position 10 μm below the sectioning plane. The cycle of sectioning imaging lasted for 21 d for the whole-brain data acquisition. The acquired EGFP-labeled neurons and their projection trajectories and targeted brain areas were built on the three-dimensional dataset of the tree shrew whole brain that consisted of 3,800 images with a voxel size of 0.32 × 0.32 × 2 μm^3^. The preprocessing of the raw dataset was carried out on a computing server. The raw images acquired within one imaging plane were stitched to form a new image at first. Then, flat-field correction was used to decrease the strip artifacts introduced by the stitching. The three-dimensional outline of the brain was extracted to remove the noise. And the format of processed images was changed to TData for rapid access for further analysis and visualization (Y. Li et al., 2017; Tan et al., 2020).

### Graph preparation

All graphs were made by using Adobe Illustrator (Adobe Software) and cellSens (Olympus Software).

## Results

### The MD projection trajectories and targeted brain regions in the tree shrew

In the first set of experiments, we attempted to identify where the FC subregions in the tree shrew received the MD projection (Wise, 2008; Carlen, 2017), which was used as the first criterion to define and outline the PFC (Divac et al., 1978). For this purpose, we injected AAV-hSyn-EGFP into the MD (Fig. 1*a*), according to the Tree Shrew (*Tupaia belangeri chinensis*) Brain in Stereotaxic Coordinates (J. N. Zhou and Ni, 2016). Following viral expression for 28 d, we used fMOST (A. Li et al., 2010; Gong et al., 2013; J. N. Zhou and Ni, 2016; Sun et al., 2019) to visualize the MD projection trajectories and targeted brain regions. We found that the MD projected out two fiber bundles, one ascending to the entorhinal cortex (Ent) of the hippocampal formation and the other ascending to the corpus striatum and then to the PFC (Fig. 1*b*). To ensure the accuracy of the injection and viral expression, we carefully checked the EGFP-labeled neurons that were in appropriate quantity and well restricted within the MD (Fig. 1*c*). These MD neurons were observed to project toward the Ent, the ventral subiculum (VS), and the lacunosum moleculare layer (LMoL) of the cornu ammonis (CA) areas of the hippocampal formation (Extended Data Fig. 1-1*a*), as well as to the caudate nucleus (Cd), internal capsule (ic), putamen (Pu), and accumbens nucleus (Acb) of the corpus striatum (Extended Data Fig. 1-1*b*). This agrees with a recent report, which suggested that the tree shrew has a primate-like structure for the corpus striatum, whereas the caudoputamen (CPu) and Acb are observed in mice or rats (Ni et al., 2018b). Furthermore, most of the fibers from the MD neurons projected across the corpus striatum to the medial, orbital, and dorsal subregions of the FC (Fig. 1*d–f*). By the MD projection definition, the mPFC and dorsal PFC (dPFC) were identified. We also observed that the frontal motor cortex (MC) did not receive the MD projection terminals (Fig. 1*g*). Using the present fMOST data and the tree shrew brain atlas (J. N. Zhou and Ni, 2016), we thus plotted the stereotaxic coordinates to determine the boundaries between the PFC and frontal MC, which range from AP (anterior–posterior) of 5.2–5.5 mm and ML (middle-lateral) of 2.10–2.58 mm (Fig. 1*d–f*, vertical pink line). Consequently, this enabled the positioning of the dPFC, mPFC, and OFC subregions in the tree shrew, as illustrated in Extended Data Figure 1-2.

Furthermore, we conducted non-transsynaptic retrograde tracing with rabies virus (RV-ΔG-N2C-EGFP) into the VS, and the result confirmed that the MD neurons directly projected to the VS neurons of the hippocampus (Extended Data Fig. 1-3). Interestingly, the tree shrew had distinct MD→VS and MD→Ent pathways, which are not present in the Mouse Connectivity Map of the Allen Brain.

### The MD projections to the L4 of the dlPFC in the tree shrew

We then conducted a second set of experiments to identify L4, which contains granular cells (smaller in size and irregular in shape) that receive the MD projection in primates, to accurately define the dlPFC. We used a Cre-dependent tracing strategy and injected the transsynaptic anterograde virus AAV1-Ef1α-Cre together with AAV-Ef1α-DIO-EYFP into the MD while AAV-Ef1α-DIO-mCherry was injected alone into the dPFC (Fig. 2*a*). Results showed that the EYFP-labeled neurons were densely distributed within the MD, which was only possible with cotransfection of both the Cre and DIO viruses. When analyzing the results, we found that the EYFP-labeled projection terminals were present in the contralateral side of the MD, relative to the injecting side (Fig. 2*b*). On the other end, most of the mCherry-labeled neurons (red) and MD projection terminals (green) were distributed in the L4 of the dPFC, fewer in the L5/6, and the least in the L2/3 (Fig. 2*c*). The presence of the mCherry-labeled neurons in the medial and orbital PC also suggested that both the areas may be the parts of the PFC. Furthermore, when looking at the L4 of the dPFC (Fig. 2*d*), the mCherry-labeled neurons were found to be densely innervated by the MD projection terminals (Fig. 2*e*), thereby confirming that the L4 of the dPFC receives the MD projection.

We reanalyzed the AP sections from the tracing study (at AP values of +7.63, +7.23, +6.83, +6.43, +6.03, and +5.76 mm) and found that the Cre receivers (mCherry-labeled neurons) of the FC subregions were distributed differently, with blue (L2/3), pink (L4), green (L5), and yellow (L6) colors (Fig. 3*a*). Counting the receivers revealed that the lateral PFC (lPFC) and dPFC had a similar cytoarchitecture with a conspicuous presence of the L4. This led us to classify them together as the dlPFC (Fig. 3*b*, top panels). The L4 of both the mPFC and OFC were distributed relatively with more receivers in the anterior parts at AP = +6.83 mm, while absent in the rearmost part at AP = +5.76 mm (Fig. 3*b*, bottom panels).

**Figure 4.**
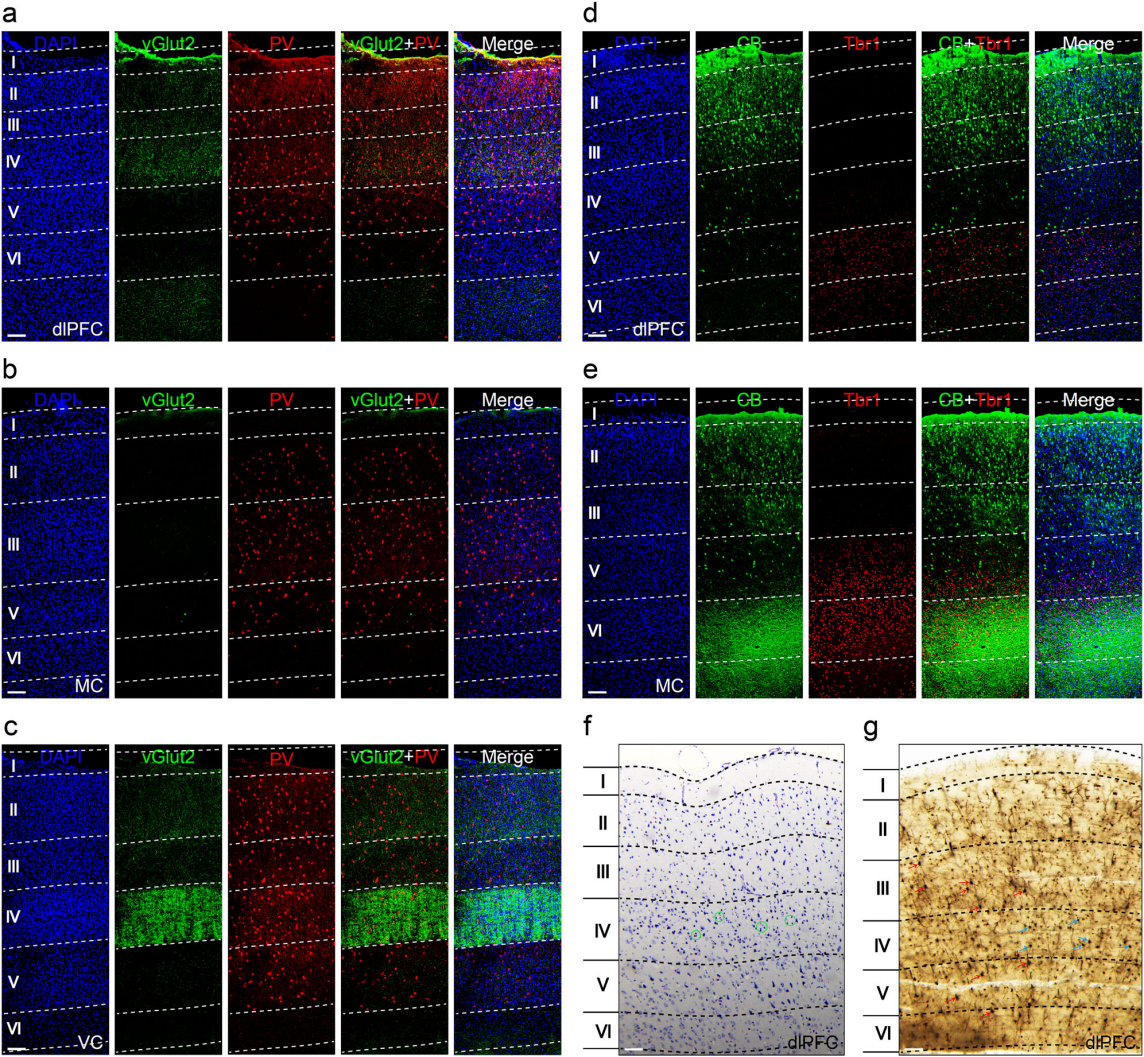
The granular dlPFC in the tree shrew. ***a***, The L4 (IV) of the dlPFC distributed with the vesicular glutamate transporter 2 (vGlut2)-positive terminals ascending from the diencephalon including the MD neurons. Many of the L4 neurons were parvalbumin (PV) positive. ***b***, The frontal motor cortex (MC) lacked the L4 and did not have the ascending vGlut2-positive terminals. The PV-positive cells distributed in the L2/3 and the L5. ***c***, The visual cortex (VC) L4 densely distributed with the ascending vGlut2-positive terminals. Many of the L4 neurons were PV positive. ***d***, ***e***, Comparison between the dlPFC and the frontal MC. The calbindin (CB)-positive cells were mainly distributed in the L2/3 of both the regions, but the CB-positive terminals were densely distributed in the L6 of the MC only. The T-box brain 1 (Tbr1)-positive cells were similarly distributed in the L5/6 of both the regions but denser in the frontal MC. ***f***, The Nissl staining of the dlPFC. The green circle indicated the granular cells (smaller in size and irregular in shape). ***g***, The Golgi staining of the dlPFC. The red arrows indicated the pyramidal cells and the blue ones suggested the granular cells. All sections were coronal with a calibration bar (white horizontal), 100 µm.

In the third set of experiments, to further clarify the granular nature of the dlPFC, we compared the cytoarchitecture of the dlPFC with the frontal MC or visual cortex (VC), which was known to be agranular or granular, respectively (Brodmann, 1909; Shipp et al., 2013). To do this, we examined the vesicular glutamate transporter 2 (vGlut2) and parvalbumin (PV) markers in the three cortices. Specifically, the vGlut2 is a biomarker for glutamatergic neurons that is strongly expressed in the diencephalon, such as the MD, and thus the vGlut2 expression can be found in the ascending projection terminals onto the L4 of the neocortex (Sarkar, 2019). We found that the vGlut2-positive terminals were present in both the dlPFC and VC, though it was much denser in the L4 of the VC. In contrast, the vGlut2 signal was absent in the frontal MC, suggesting the lack of the L4 (Fig. 4*a–c*, vGlut2 panels). On the other hand, the PV marker, which is known to be a biomarker for a type of GABAergic neurons, which is preferentially expressed in the middle layer (L3–4) of the neocortex, showed that many of the neurons in the L4 were PV positive in the dlPFC and VC, while the L3 of the frontal MC was enriched with PV-positive neurons (Fig. 4*a–c*, PV panels).

Calbindin (CB) is predominantly found in the L2/3 of the neocortex. As predicted, we observed a similar distribution of CB-positive neurons in the L2/3 of both the dlPFC and frontal MC. However, the CB-positive terminals were found in the L6 of the frontal MC but not in the dlPFC (Fig. 4*d*,*e*, CB panels). Also, T-box brain 1 (Tbr1) is a transcription factor known to be expressed in the postmitotic projection neurons. We found that Tbr1 was present in the L5/6 neurons of the dlPFC, with a pattern that was like that of the frontal MC, though with a denser signal in the later (Fig. 4*d*,*e*, Tbr1 panels).

Relative to the larger pyramidal cells, the granular cells found in the L4 are smaller in size and irregular in shape, and hence they are typically identified using the Nissl or Golgi staining. Here, we conducted both the stainings and validated the presence of granular cells in the L4 of the dlPFC in the tree shrew (Fig. 4*f*,*g*), suggesting that the tree shrew has evolved a granular dlPFC.

### The dlPFC connections with the MD reciprocally in the tree shrew

Since the MD was found to project to the L4 of the dlPFC, we then aimed to investigate if and how the dlPFC projected back to the MD, which would be the third criterion for defining the dlPFC. To accomplish this, we injected the transsynaptic retrograde virus AAVretro-hSyn-GFP-Cre-wpre-pA into the dlPFC to express both GFP and Cre, while AAV-Ef1α-DIO-mCherry-wpre-pA was injected into the MD (Extended Data Fig. 2-1*a*) as the MD projection neurons could acquire the Cre retrogradely. We observed that the GFP-labeled neurons were mainly distributed in the L5/6 of the dlPFC (Extended Data Fig. 2-1*b*). On the other end, the mCherry-labeled neurons (red) that required expression of both the DIO and Cre were mainly distributed in the MD, with fewer present in the paracentral nucleus (PC) of the thalamus, which was affiliated with the GFP-labeled dlPFC-projection terminals (Extended Data Fig. 2-1*c*).

We subsequently injected the combination of AAV-hsyn-FLExFRT-Mgfp-2A-Synaptophysin-mRuby-WPRE-pA and AAV-EF1α-FLP-WPRE-Pa helper virus into the dlPFC (Extended Data Fig. 2-1*d*). As a result of the helper virus, Mgfp (green) would be expressed in the cell body but synaptophysin enabled mRuby (red) to be transported to the dlPFC projection terminals. We found that Mgfp and mRuby (merged as yellow) were mainly observed in the L5, while a fewer number was in the L3/4 or L6 of the dlPFC (Extended Data Fig. 2-1*e*). We determined that mRuby was denser than Mgfp in the dlPFC projection terminals in the MD and PC (Extended Data Fig. 2-1*f*). Altogether, it was determined that the dlPFC and MD have the connections to one another. Notably, the L5/6 of the dlPFC projected back to the MD.

**Figure 5.**
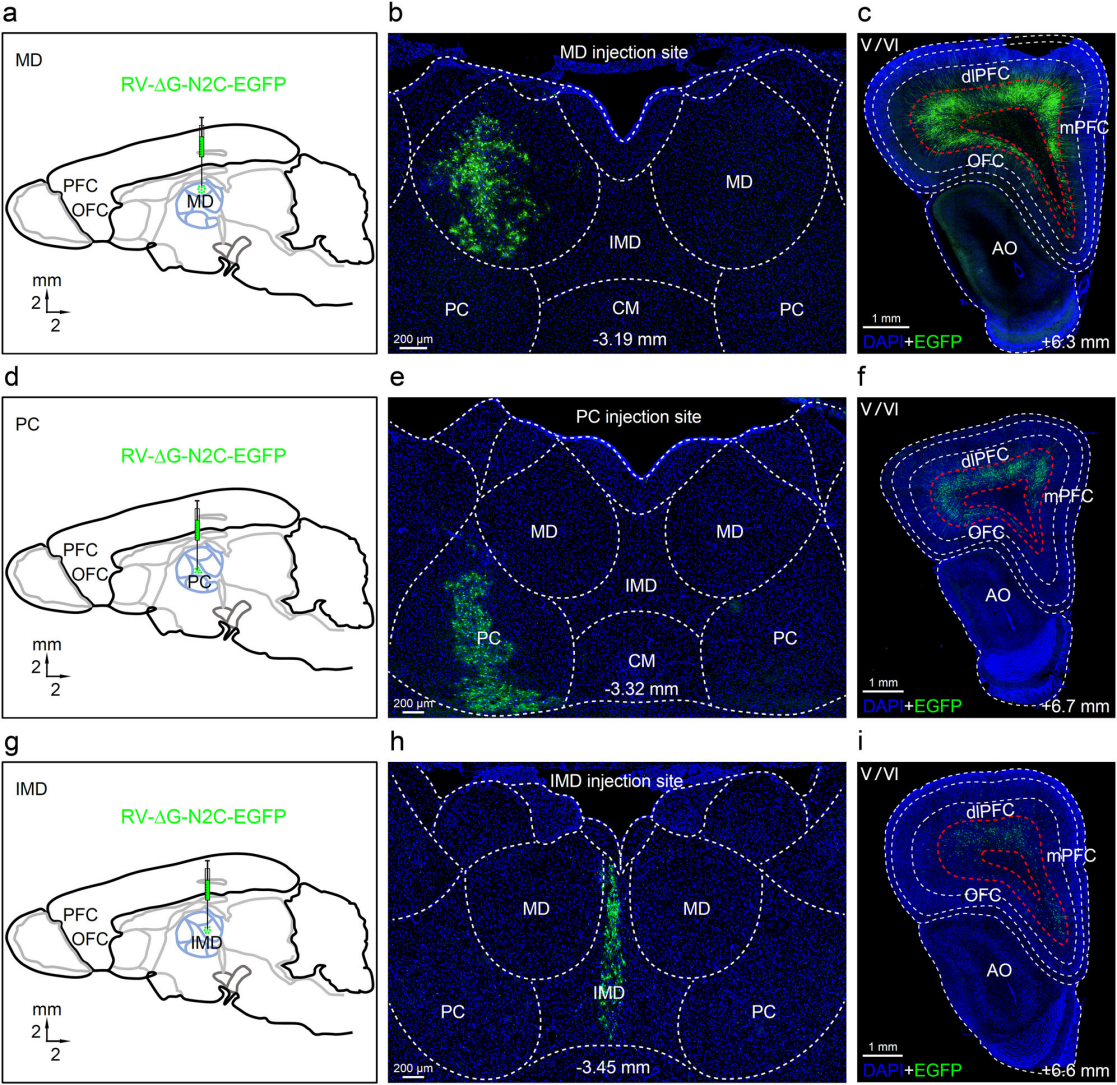
Organization of the dlPFC neurons projecting to the thalamic nuclei. ***a–c***, From the sagittal view of the tree shrew brain, RV-ΔG-N2C-EGFP was injected into the MD. The EGFP-labeled neurons in the MD were the receivers, from which retrograde tracing visualized the starters that were the EGFP-labeled neurons densely located in the L5/6 of the dlPFC and the mPFC, and fewer in the OFC. ***d–f***, RV-ΔG-N2C-EGFP was injected into the paracentral nucleus (PC). The EGFP-labeled neurons in the PC were the receivers, from which retrograde tracing visualized the starters that were the EGFP-labeled neurons located in the L5/6 of the dlPFC and the mPFC, and few in the OFC. ***g–i***, RV-ΔG-N2C-EGFP was injected into the intermediodorsal nucleus (IMD). The EGFP-labeled neurons in the IMD were the receivers, from which retrograde tracing visualized the starters that were the EGFP-labeled neurons sparsely located in the L5/6 of the dlPFC and the mPFC, and very few in the OFC. CM, central medial nucleus; AO, anterior olfactory nucleus.

To illuminate the specifics, we utilized the non-transsynaptic retrograde tracing rabies virus RV-ΔG-N2C-EGFP and injected it into the MD, PC, and intermediodorsal nucleus (IMD), respectively. Upon injection of the non-transsynaptic retrograde tracing rabies virus RV-ΔG-N2C-EGFP into the MD (Fig. 5*a*) over a 7 d period, the EGFP-labeled neurons were observed in the MD (Fig. 5*b*). These MD neurons were receivers, through which retrograde tracing identified the starters densely located in the L5/6 of the dlPFC and mPFC, and with fewer numbers in that of the OFC (Fig. 5*c*). Once the non-transsynaptic retrograde tracing rabies virus RV-ΔG-N2C-EGFP was injected into the PC (Fig. 5*d*) and exposed for a duration of 7 d, the EGFP-labeled neurons were dispersed in the PC (Fig. 5*e*). Through retrograde tracing, it was determined that the starters were in the L5/6 of the dlPFC and mPFC and in fewer numbers in that of the OFC (Fig. 5*f*). Upon waiting 7 d after injecting the non-transsynaptic retrograde tracing rabies virus RV-ΔG-N2C-EGFP into the IMD (Fig. 5*g*), we established that the EGFP-labeled neurons were in the IMD (Fig. 5*h*). Retrograde tracing disclosed that the starters sparsely distributed in the L5/6 of the dlPFC and mPFC and with the fewest numbers in the OFC (Fig. 5*i*).

The coronal sections in the AP coordinates range of +7.10 to +4.09 mm of the tracing study showed the starters that artificially were colored by yellow (dlPFC), pink (mPFC), and blue (OFC), according to injection sites of the retrograde tracing virus into the MD (Extended Data Fig. 2-2*a*), PC (Extended Data Fig. 2-2*b*), and IMD (Extended Data Fig. 2-2*c*), respectively. The neuronal numbers count suggested that the starters projecting to the MD, PC, and IMD were mainly originating from the L5/6 of the dlPFC, fewer from the mPFC, and the least from the OFC (Extended Data Fig. 2-2*d–f*).

Hence, it is evident that the L5/6 of the dlPFC project to the MD. Moreover, the dlPFC and mPFC share similar projections to the MD, PC, and IMD of the thalamus.

### Comparison between the dlPFC and mPFC in reciprocal connections with the MD

The murine is characterized by the mPFC, OFC, and dorsolateral part of the FC, which are all agranular, and the former two meet the MD projection criterion of the PFC. In marked contrast, in the tree shrew, the mPFC, OFC, and dlPFC are all granular or dysgranular, where the L4 receives the MD projection and the L5/6 projects back to the MD.

The evolutionary sketch of the PFC is not fully clear yet. However, it is interesting that there is only the agranular mPFC in the murine species, the granular mPFC and dlPFC in the tree shrew, and the granular mPFC, dlPFC, and orbital PFC in most primates. Thus, it is interesting to evaluate the differences in the projection of the mPFC and dlPFC to thalamic nuclei. Therefore, we injected AAV-hsyn-mCherry-wpre-pA and AAV-hsyn-EGFP-wpre-pA, respectively, into the dlPFC and mPFC (Extended Data Fig. 3-1*a*). After 25 d, we observed that while both regions shared similar projections to the MD, PC, and IMD, only the dlPFC projected to the central medial nucleus (CM) and submedius nucleus (Sub; Extended Data Fig. 3-1*b*).

The injection of AAV-hSyn-GFP-wpre-pA into the mPFC (prelimbic cortex, PrL) resulted in the labeling of the L2–6 neurons, including the dysgranular L4 with few granular cells located at the AP = +6.70 mm (Extended Data Fig. 3-1*c*). This was accompanied by the distribution of the GFP-labeled projection terminals to the MD, PC, and IMD (Extended Data Fig. 3-1*d*), which were presumed to originate from the L5/6 neurons of the mPFC. Likewise, AAV-hsyn-mCherry-wpre-pA was injected into the more posterior site of the mPFC (AP = +5.16 mm) that showed typical granular L4 (Extended Data Fig. 3-1*e*), along with the mCherry-labeled projection terminals mainly targeting the PC (Extended Data Fig. 3-1*f*). In contrast, the injection of AAV-hSyn-mCherry-wpre-pA into the dlPFC (AP = +7.23 mm) revealed dense granular cells in the L4 (Extended Data Fig. 3-1*g*), while the mCherry-labeled terminals from the L5/6 of the dlPFC largely projected to the MD, PC, IMD, CM, and Sub (Extended Data Fig. 3-1*h*).

**Figure 6.**
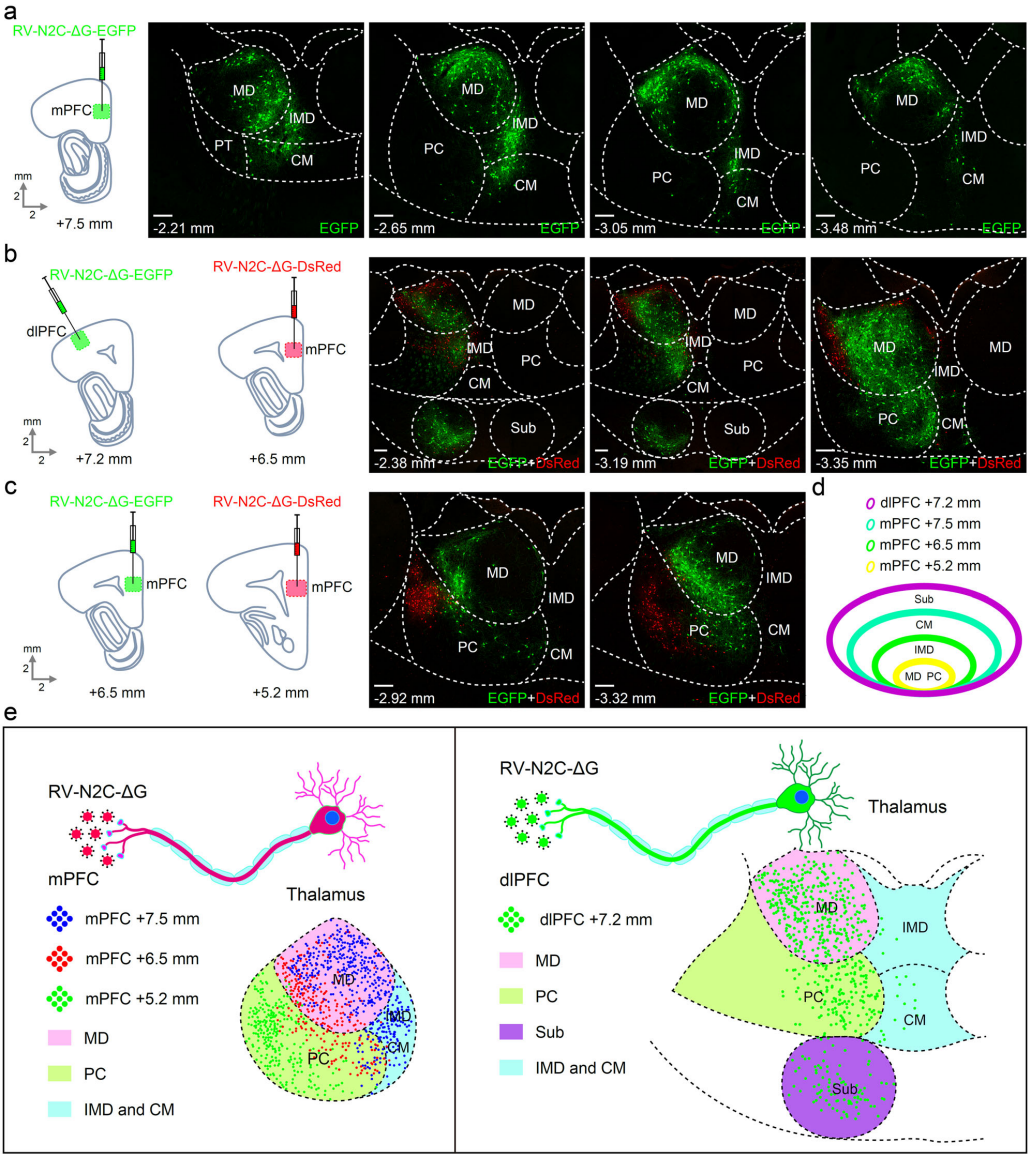
Organization of the thalamic neurons projecting to the dlPFC and the mPFC. ***a***, The non-transsynaptic retrograde tracing virus RV-N2C-ΔG-EGFP was injected into the mPFC, and the EGFP-labeled neurons were found in the MD, IMD, and CM of the thalamus at the AP coordinates from −2.21 to −3.48 mm, suggesting that these EGFP-labeled neurons in the thalamus projected to the mPFC. ***b***, The RV-N2C-ΔG-EGFP and RV-N2C-ΔG-DsRed were injected into the dlPFC and the mPFC, respectively. The EGFP-labeled neurons were found in the MD, PC, and Sub, but sparsely also in the CM. The DsRed-labeled neurons were found in the MD, PC, CM, and IMD. These labeled neurons were projected to the dlPFC (EGFP) and the mPFC (DsRed), respectively. ***c***, The RV-N2C-ΔG-EGFP and RV-N2C-ΔG-DsRed were injected into the mPFC at the AP coordinates +6.5 or +5.2 mm. EGFP-labeled neurons were found in the MD and PC. The DsRed-labeled neurons were found only in the PC. ***d***, Summary of the thalamic projection areas to the dlPFC or the mPFC. The MD and PC are the common areas projected to both the dlPFC and the mPFC. ***e***, The topographic distribution of the thalamic projecting neurons to the different AP coordinates of the dlPFC and the mPFC. Scale bar, 200 μm. MD, mediodorsal nucleus; PC, paracentral nucleus; Sub, submedius nucleus; IMD, intermediodorsal nucleus; CM, central medial nucleus.

Next, a different approach was taken to analyze the afferent projections received by the mPFC and dlPFC. We injected the non-transsynaptic retrograde tracing rabies virus RV-N2C-ΔG-EGFP or RV-N2C-ΔG-DsRed into dlPFC or mPFC, or both. This retrograde tracing revealed that neurons from the MD, PC, IMD, and CM of the thalamus projected to the mPFC (PrL). The distribution of the neurons within these thalamic nuclei displayed a topographic pattern corresponding to the AP coordinates (−2.21, −2.65, −3.05, and −3.48 mm) (Fig. 6*a*). Notably, the EGFP-labeled neurons were mainly located in the rim of the MD at the AP coordinates.

We then injected the non-transsynaptic retrograde tracing rabies virus RV-N2C-ΔG-EGFP into the dlPFC and the RV-N2C-ΔG-DsRed into the mPFC of the same animals, respectively. The EGFP-labeled neurons that projected to the dlPFC were mostly located in the center of the MD, in stark contrast to the DsRed-labeled neurons that projected to the mPFC which were mainly observed in the rim of the MD (Fig. 6*b*). In addition, the EGFP-labeled neurons were also located in the Sub which projected to the dlPFC. Furthermore, the non-transsynaptic retrograde tracing rabies virus RV-N2C-ΔG-EGFP and RV-N2C-ΔG-DsRed were injected into the mPFC at different APs (+6.5 or +5.2 mm). The retrograde tracing showed that the EGFP-labeled neurons were mostly observed in the MD and the DsRed-labeled neurons were mainly identified in the PC, projecting to the anterior (+6.5 mm) or posterior (+5.2 mm) parts of the mPFC, respectively (Fig. 6*c*). Thus, it can be concluded that the mPFC and dlPFC have shared projections from the MD, PC, IMD, and CM, with the dlPFC also receiving projections from the Sub (Fig. 6*d*,*e*).

By combining the findings of our experiments, we observed reciprocal connections of the mPFC and dlPFC with the MD, PC, IMD, and CM. Additionally, the dlPFC had reciprocal connections with the Sub.

## Discussion

**Figure 7.**
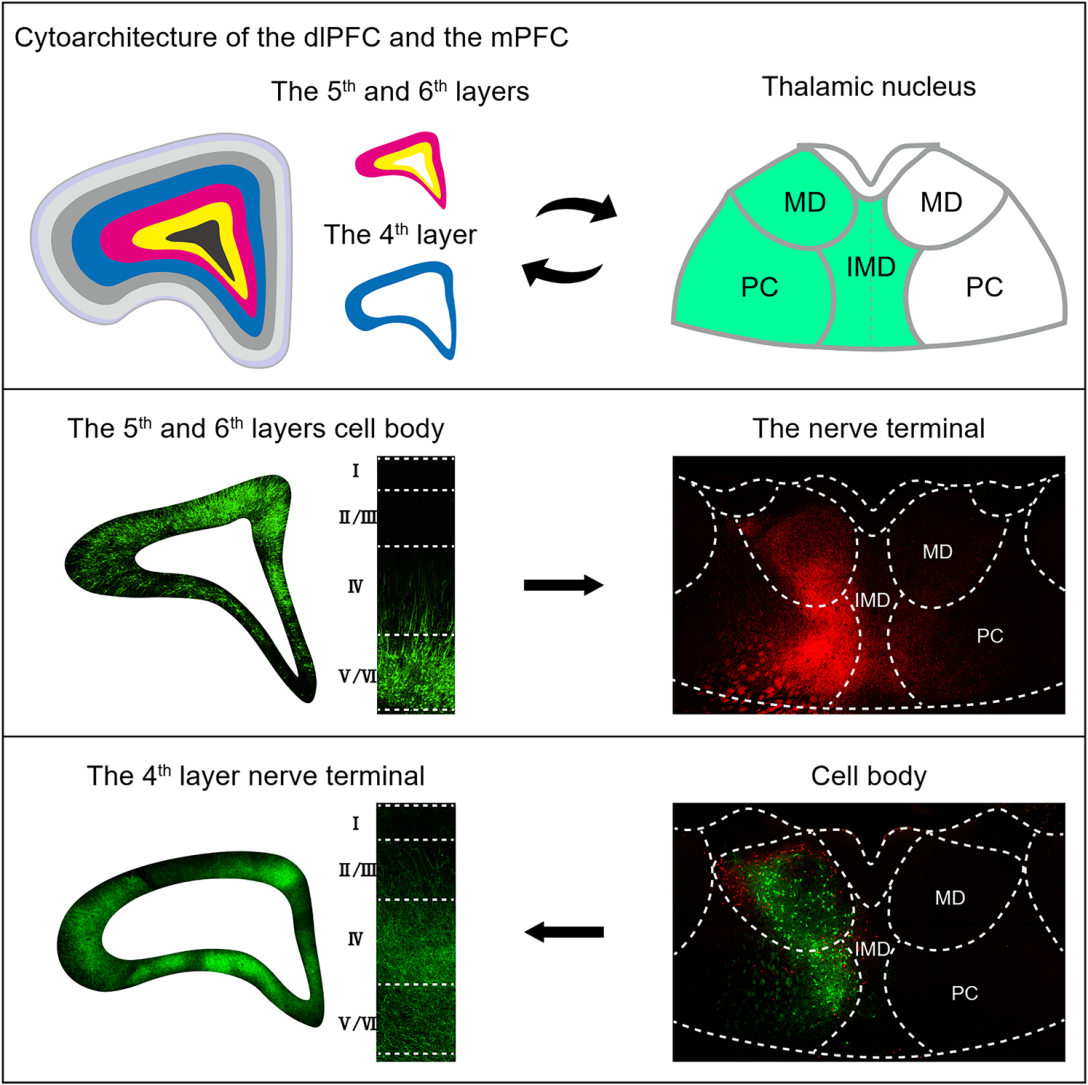
Summary of the granular dlPFC→MD in the Chinese tree shrew. This study found that the Chinese tree shrew has evolved the granular dlPFC with reciprocal connections with the MD. MD, mediodorsal nucleus; PC, paracentral nucleus; IMD, intermediodorsal nucleus; CM, central medial nucleus.

The Chinese tree shrew is the closest now-living sister of primates (Y. Fan et al., 2013) and has remarkably evolved a primate-like dlPFC that contains an L4 enriched with granular cells, with established MD→PFC reciprocal connections (Fig. 7). This is significantly different from the agranular mPFC, OFC, and dorsolateral FC of the murine, which only partly satisfy the MD→PFC definition (Leonard, 1969; 2016; Guldin et al., 1981). These remarkable differences could implicate that the tree shrew is an alternative for researching higher cognitive functions (Savier et al., 2021) and related neuropsychiatric disorders (Y. Zhou et al., 2015).

### The dlPFC contributions to emotion-related higher cognitive functions

The MD projection definition of the PFC across species continues to be highly debated (Carlen, 2017), as the FC subregions receiving the MD projections are granular in primates, but agranular in the murine (Lein et al., 2007). In this study, we assessed the dlPFC of the tree shrew using the strictest criteria, by which we found that the tree shrew had evolved a primate-like dlPFC with an L4 enriched with granular cells. The L4 received the MD projections while the L5/6 sent projections back to the MD. Furthermore, based on the tree shrew brain atlas (J. N. Zhou and Ni, 2016) from a previous study and the fMOST data in the present study, we provided a stereotaxic-coordinate map for positioning the dlPFC, mPFC, and OFC subregions (Extended Data Fig. 1-2).

It is critical to investigate further the capacities of the dlPFC of the tree shrew in higher cognitive functions. Tree shrews have been observed to demonstrate satisfactory results in the spatial delayed alternation task in T-maze, which is impaired by the lesion of the frontal pole (Passingham, 1978), where it is defined as the dPFC in the present study, to possibly contribute to this cognitive performance. Furthermore, tree shrews have proved to be inferior to monkeys while performing the Wisconsin General Test Apparatus working memory task (Ohta et al., 1985; Cai et al., 1993).

On the other hand, tree shrews may be particularly suited for studying stress-related cognitive dysfunctions that are observed in many neuropsychiatric disorders such as MDD. Recent research has demonstrated that the corticotrophin-releasing hormone (CRH) in activating the hypothalamic-pituitary-adrenal (HPA) of the tree shrew responds to stress similarly to how it does in humans (L. Fang et al., 2016), indicating a possible vulnerability to stress. This vulnerability could be attributed to a unique active glucocorticoid receptor response element in the CRH promoter region that leads to higher CRH expression (L. Fang et al., 2016). Furthermore, experiments have documented evidence of CA1/3 neuronal atrophy in tree shrews as a result of psychosocial conflict (Fuchs et al., 1995; Gould et al., 1997), as well as reductions of dendrite complexity and spine density in the PFC neurons due to chronic uncontrollable stress (R. J. Liu and Aghajanian, 2008). These deficits are hypothesized to be the cause of MDD in humans (Duman and Aghajanian, 2012; Richter-Levin and Xu, 2018), as stress has been known to weaken the functions of the dlPFC in primates and humans (Arnsten, 2015). Thus, tree shrews make an ideal animal model for studying these effects since exhibiting stable MDD-like symptoms in response to psychologic stress, which can be alleviated by using the clinically available antidepressants (Fuchs et al., 1996; Wang et al., 2013a).

It has been stated that the OFC of the tree shrew can be considered as the orbital PFC, which meets the definition of the MD→PFC projection as proposed by Rose and Woolsey (1948). However, the reciprocal connections with the MD are not as dense as those found in the mPFC and dlPFC. In addition, the L4 of the OFC appears to be thinner and has fewer granular cells than the mPFC or dlPFC. As a result, the OFC may not have been evolved as well as the dlPFC or mPFC in the tree shrew. This could be an explanation for why tree shrews are inferior to monkeys when performing working memory tasks (Ohta et al., 1985; Cai et al., 1993), because all subregions of the PFC are believed to be integrated for processing higher cognitive functions (Goldman-Rakic, 1990; Fuster, 2001; Carlen, 2017). Other possibilities cannot be ruled out as the OFC is not a central part of working memory networks in primates (Constantinidis and Procyk, 2004).

### The physiology and anatomy features of the tree shrew

Tree shrews are widely accepted to be related to primates due to their similar features in terms of both physical anatomy and genetic makeup. In the terms of physical appearance, tree shrews generally have longer snouts and larger eyes than primates, and they also possess claws rather than nails. Additionally, previous research on the connections between tree shrews and primates has revealed that both may belong to the Euarchonta clade or that tree shrews are the closest living sister of primates (Y. Fan et al., 2013). Moreover, studies have suggested that a brain region in tree shrews known as the corpus striatum has shown a primate-like structure. Here, we find that the MD projects to several parts of the hippocampal formation and the primate-like corpus striatum. These two brain regions are also known to be critical for higher cognitive functions.

This innovative research on the Chinese tree shrew suggests that the dlPFC has developed the granular L4 with reciprocal connections with MD, which also has sent the connections to the hippocampus and striatum, marking a primate-like neurocircuit. To our knowledge, it is for the first time that we defined the dlPFC of the tree shrew, which is highly consistent with the original taxonomy of tree shrews in the 1920s (Buettner-Janusch, 1963; Martin, 1968; Clark, 1971). Furthermore, this quality is presumed to be particularly advantageous for the Chinese tree shrew, as the dlPFC can handle the various information from the thalamus while giving feedback to the MD. Moreover, the MD also projects to the hippocampal formation and corpus striatum, both of which are known to play a vital role in higher cognitive functions. Thus, this discovery provides the proof that the dlPFC of the tree shrew is likely to have established a hierarchical neurocircuit among these brain regions to be comparable with that of primates, implying that it can perform more difficult mental representations and executions (Goldman-Rakic, 1990; Arnsten, 2015; Carlen, 2017).

## Synthesis

Reviewing Editor: Matthew Grubb, King's College London Decisions are customarily a result of the Reviewing Editor and the peer reviewers coming together and discussing their recommendations until a consensus is reached. When revisions are invited, a fact-based synthesis statement explaining their decision and outlining what is needed to prepare a revision will be listed below. The following reviewer(s) agreed to reveal their identity: Samantha White, Jerome Sallet. Note: If this manuscript was transferred from JNeurosci and a decision was made to accept the manuscript without peer review, a brief statement to this effect will instead be what is listed below.

Synthesis:

Both Reviewers are very positive about the manuscript, but suggest important changes to the text in order to clarify the claims being made. Please make sure you address all of their comments below in a fully revised manuscript.

Reviewer 1:

Summary and impression:

This is a well-done anatomical study investigating a granular prefrontal cortical area in a representative tree shrew species, Tupaia belangeri Chinensis. The authors provide high quality data showing connectivity between the mediodorsal thalamus (MD), hippocampus, corpus striatum, and dorsolateral prefrontal cortex (dlPFC). They highlight the reciprocal connections between MD and dlPFC, specifically layer IV, and suggest this connectivity resembles the primate dlPFC. The figures are clear and the motivation and results of the study are well presented. My concerns with the study are related to statements made about the tree shrew dlPFC and how it ultimately relates to the dlPFC of primate species. Below I have referred to specific instances that I think need to be addressed.

1) I think the term 'primates-specific' as it is used to describe the tree shrew dlPFC should be changed to something more broad, for instance, 'primate-like'. This change in terminology will reduce potential confusion that the dlPFC in tree shrews and primates are homologous. Based on a lack of evidence for dlPFC (area 46) in other non-simian primate species (see Preuss and Wise, 2022), these areas evolved independently in tree shrews and simian primates. The authors don't directly suggest these regions across species are homologous, but I think care should be taken to avoid unassuming readers from coming to that conclusion on their own.

2) In the final paragraph of the section: 'The MD projects to the L4 of the dlPFC in the tree shrew', the authors state '...'suggesting that the tree shrew has evolved the granular dlPFC'. This is a small point, but 'the' should be replaced with 'a'. I make this suggestion to again, reduce likelihood that a homology is presumed.

3) In the section 'Comparison between the dlPFC and mPFC in reciprocal connections with the MD', second paragraph, the authors begin with 'The evolutionary sketch of the PFC involves a gradual progression ...' I think that this statement is problematic because it implies that the dlPFC as represented was present in the last common ancestor of tree shrews and primates. As stated in the first point, there is no known evidence that non-simian primates have granular dlPFC, which leaves a major gap in the family tree for the existence of this area (see Preuss and Wise, 2022). Without that evidence, it's likely these areas evolved convergently and I think this point should be noted. The fact that these areas are convergent does not undermine the anatomy presented in the manuscript, and does not undermine the utility of tree shrews for neuroscientific studies. It simply makes the relationship between species more defined and could explain why similarities and differences in future studies of dlPFC in tree shrews vs. primates.

4) In the first paragraph of the discussion, the authors state 'These remarkable differences provide us with a unique opportunity to understand the evolutionary ...'. I think there are several strengths of this study, and the authors provide a number of reasons for the utility of the work (including role of this region for higher order function, accessibility of the research model, etc), but I don't believe this study on it's own contributes to the evolutionary understanding of PFC in primates. It may provide evidence of a convergent dlFPC in a sister species, but without the evidence stated in my earlier points, this study cannot lead to conclusions about primate evolution. I think the authors should change this statement to reflect other strengths of the work.

5) In the section titled 'Phylogenetic origin of the dlPFC in the tree shrew' the authors open the second paragraph with the statement 'This innovative research on the Chinese tree shrew confirms...'. As I've stated, I think this study provides compelling evidence for a granular dlPFC in tree shrews, however, I don't think the authors provide enough evidence to support the emergence of dlPFC in the last common ancestor of Euarchontans. The rest of the paragraph summarizes the study nicely, and therefore I think the authors should remove this first statement.

6) In the methods, the authors mention adult tree shrews over 1.5 years are used. What is the lifespan of a tree shrew? Given the PFC is one of the final cortical regions across species to mature, was there any concern that the PFC was not fully mature by the time these tree shrews were studied?

Reviewer 2:

In this study the authors are describing the presence of a dorsal frontal granular cortical region that receives projection from the mediodorsal thalamic nucleus in the tree shrew.

While the main focus of the study is to highlight the presence of a so-called primate-specific feature in a non-human primate brain, I would like to suggest to the authors that some evidence should be presented in a different order.

Following their identification of MD projections to the frontal cortex, the authors might want to present results about the distribution of the granular areas in the frontal cortex. Such results are currently only presented in Fig3. Furthermore, Fig 3f and G are difficult to read due to their resolution. In addition, Supp Fig4 could be included in the main text, to further strengthen the argument that the presence of a granular frontal cortex (and of several areas) in tree shrew brain is clearly distinct to what could be observed in a murine brain. Finally, as in primates, the MD projections appear to be topographically organized (see Goldman-Rakic and Porrino 1985).

Introduction:

OFC in rodents is mPFC in primates (p3, 1st paragraph). I am not sure about this claim. The authors might want to delete it or rephrase it.

Moreover, what do the authors mean by "although the OFC could potentially also be a part of the PFC" (p5, last paragraph)?

Discussion:

The authors might want to add a reference regarding the link between OFC in primates and in tree shrew (p14, 2nd paragraph).

The authors linked the thinner layer 4 in OFC to differential performance in working memory in primates vs tree shrew. However, the OFC is not a central part of the working memory networks in primates (see Constantinidis and Procyk 2004). In addition, the authors might want to quote that DPF lesion in tree shrew is impacting on their performance in a delayed alternation spatial task (Passingham 1978), but that no frontal region in tree shrew shares functional similarities with primate frontal eye field (Rempel et al. 2006).

Methods:

How many animals and of which sex were included in this study ?
