# Supplementary figures and images for: Depicting Primate-Like Granular Dorsolateral Prefrontal Cortex in the Chinese Tree Shrew
Source: eNeuro. 2024 Oct 24;11(10):ENEURO.0307-24.2024. doi: 10.1523/ENEURO.0307-24.2024 (PMC11514722; doi:10.1523/ENEURO.0307-24.2024)

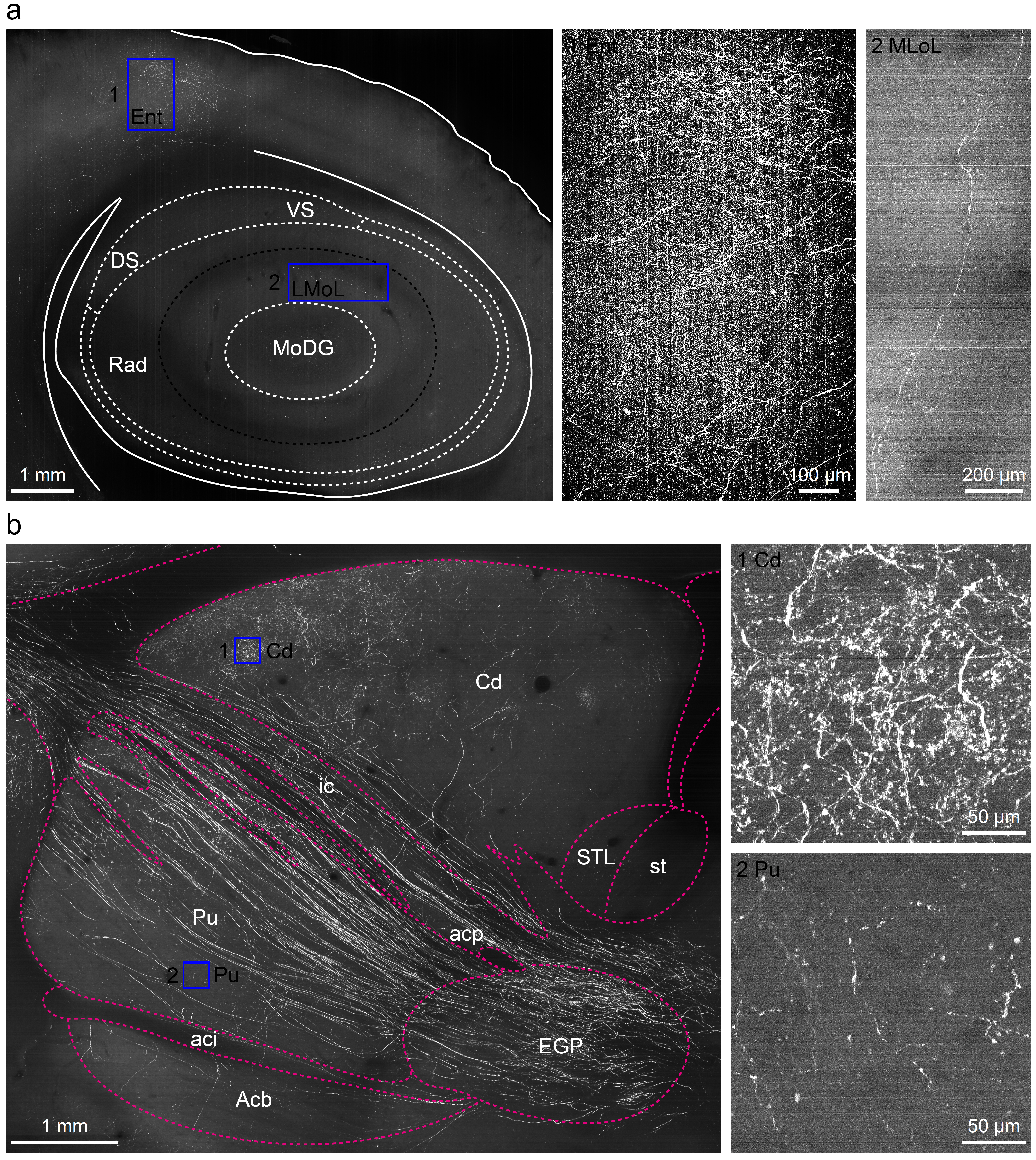

Supplement: Figure 1-1 — The MD neurons project to the entorhinal cortex (Ent) and the cornu ammonis (CA) areas of the hippocampal formation, and to the primates-like corpus striatum. (a) The EGFP-labelled neurons within the MD project to the Ent and the lacunosum molecular layer (LMoL) of the CA areas. (b) The EGFP-labelled neurons within the MD also project to the corpus striatum, which developed to be a primates-like structure. VS = ventral subiculum; DS = dorsal subiculum; MoDG = molecular layer of the dentate gyrus (DG). Cd = caudate nucleus; Pu = putamen; ic = internal capsule; acp = anterior commissure posterior part; STL = bed nucleus of the stria terminalis lateral division; st = stria terminalis; aci = anterior commissure intrabulbar part; Acb = accumbens nucleus; EGP = external globus pallidus. Download Figure 1-1, TIF file. [file eneuro-11-ENEURO.0307-24.2024-s001.tif]

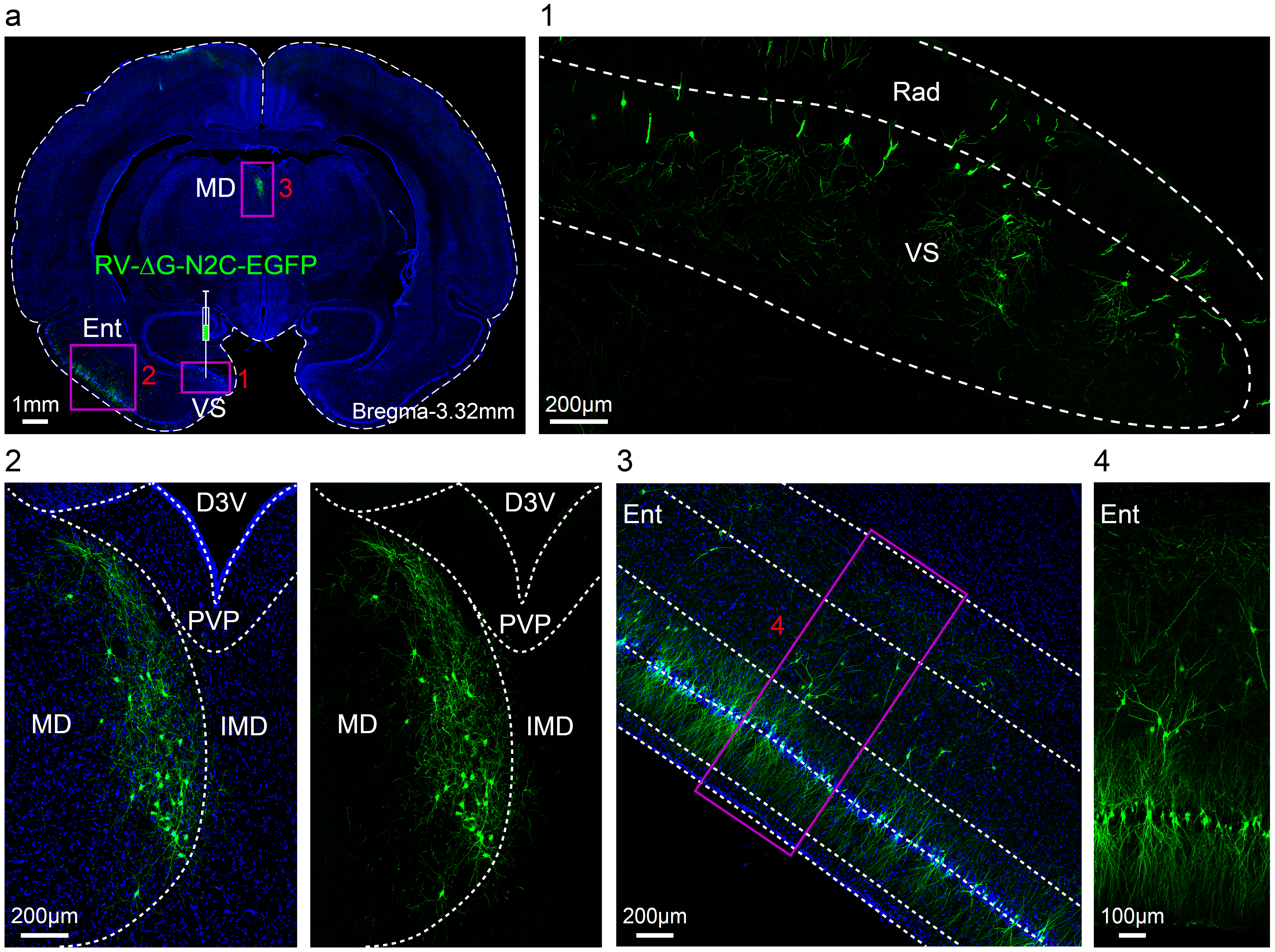

Supplement: Figure 1-3 — Non-transsynaptic retrograde tracing virus RV-ΔG-N2C-EGFP confirms direct projections from the mediodorsal nucleus (MD) to the ventral subiculum (VS) of the hippocampus. (a) RV-ΔG-N2C-EGFP was injected into the ventral subiculum (VS). (1) The injection site in the VS. (2) Neurons were labeled within the MD with (left) or without DAPI (right) signal, suggesting that the MD projects directly to the VS. (3-4) The neurons in the L2/3 of the entorhinal cortex (Ent) were also labeled with (3) or without DAPI (4) signal, suggesting that the Ent L2/3 directly projects to the Vs. Rad = radiatum layer of the CA areas. D3 V = third ventricle; PVP = paraventricular nucleus posterior part; IMD = intermediodorsal nucleus. Download Figure 1-3, TIF file. [file eneuro-11-ENEURO.0307-24.2024-s003.tif]

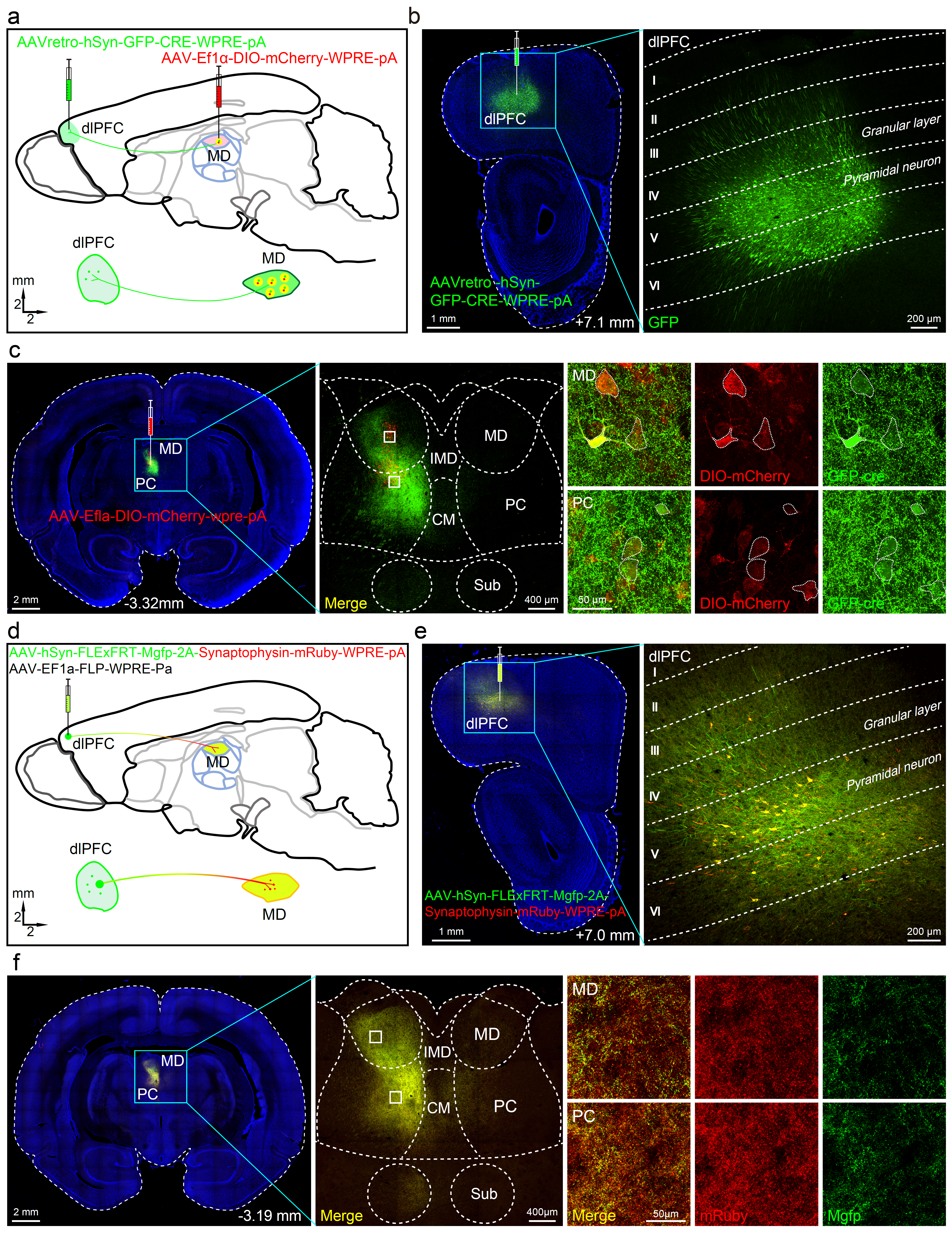

Supplement: Figure 2-1 — Reciprocal connections between the dlPFC and the MD. (a) AAVretro-hSyn-GFP-Cre-wpre-pA was injected into the dlPFC and AAV-Ef1α-DIO-mCherry-wpre-pA was injected into the MD. (b) The GFP-labelled neurons were observed in the 4-6 layers of the dlPFC, where the expressed Cre can be retrogradely transferred to the projection neurons of the MD. (c) The retrograde Cre then drove mCherry expression (AAV-Ef1α-DIO-mCherry-wpre-pA) in the MD and paracentral nucleus (PC), indicating that these mCherry-labelled neurons projected to the GFP-labelled neurons in the dlPFC. Conversely, the GFP-labelled neurons sent projection terminals to surround the mCherry-labelled neurons in the MD. (d) AAV-hsyn-FLExFRT-Mgfp-2A-Synaptophysin-mRuby-WPRE-pA and AAV-EF1α-FLP-WPRE-Pa helper were injected into the dlPFC. (e) Both Mgfp (Green) and mRuby (Red) labeled neurons were observed in the 4-6 layers of the dlPFC. (f) The dlPFC-projection terminals showed denser mRuby than Mgfp signals in the MD and PC. IMD = intermediodorsal nucleus; CM = central medial nucleus; Sub = submedius nucleus. Download Figure 2-1, TIF file. [file eneuro-11-ENEURO.0307-24.2024-s004.tif]

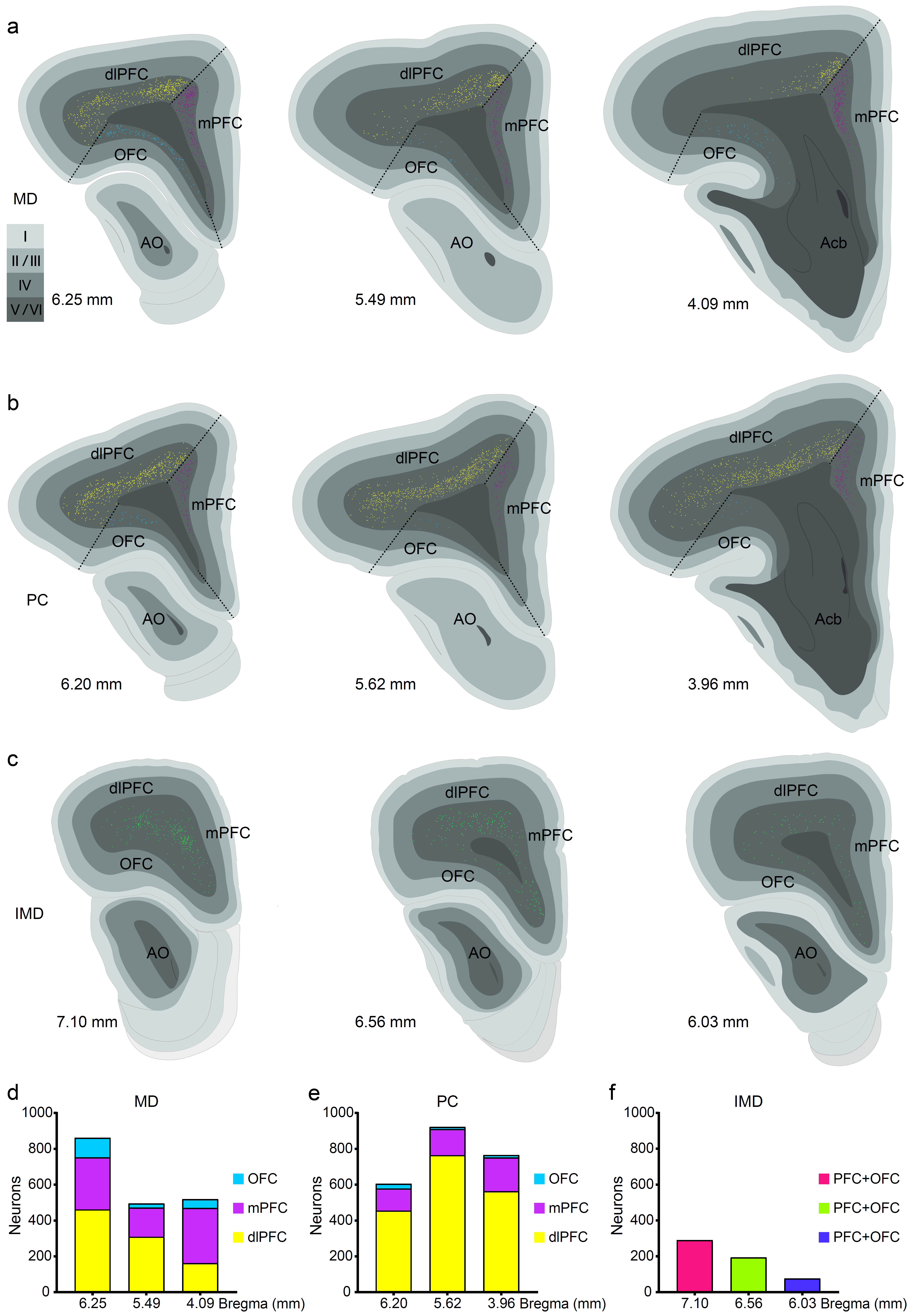

Supplement: Figure 2-2 — Neuronal numbers project to the MD, PC, and IMD. (a-c) The topographical distribution of neuronal numbers in the dlPFC, mPFC, and OFC projected to the MD, PC, and IMD. The colors represented neurons (each dot = a neuron) in the dlPFC (yellow), mPFC (red), and OFC (green). (d) From the AP +6.25 to +4.09 mm indicating neuronal numbers projected to the MD, which were increasing in the mPFC while decreasing in the dlPFC and OFC. (e) Following the AP from +6.25 to +4.09 mm for neurons projecting to the PC, neuronal numbers of the mPFC, dlPFC, and OFC remained unchanged. (f) As the AP at +7.1 to +6.03 mm, neuronal numbers projecting to the IMD were decreasing. Download Figure 2-2, TIF file. [file eneuro-11-ENEURO.0307-24.2024-s005.tif]

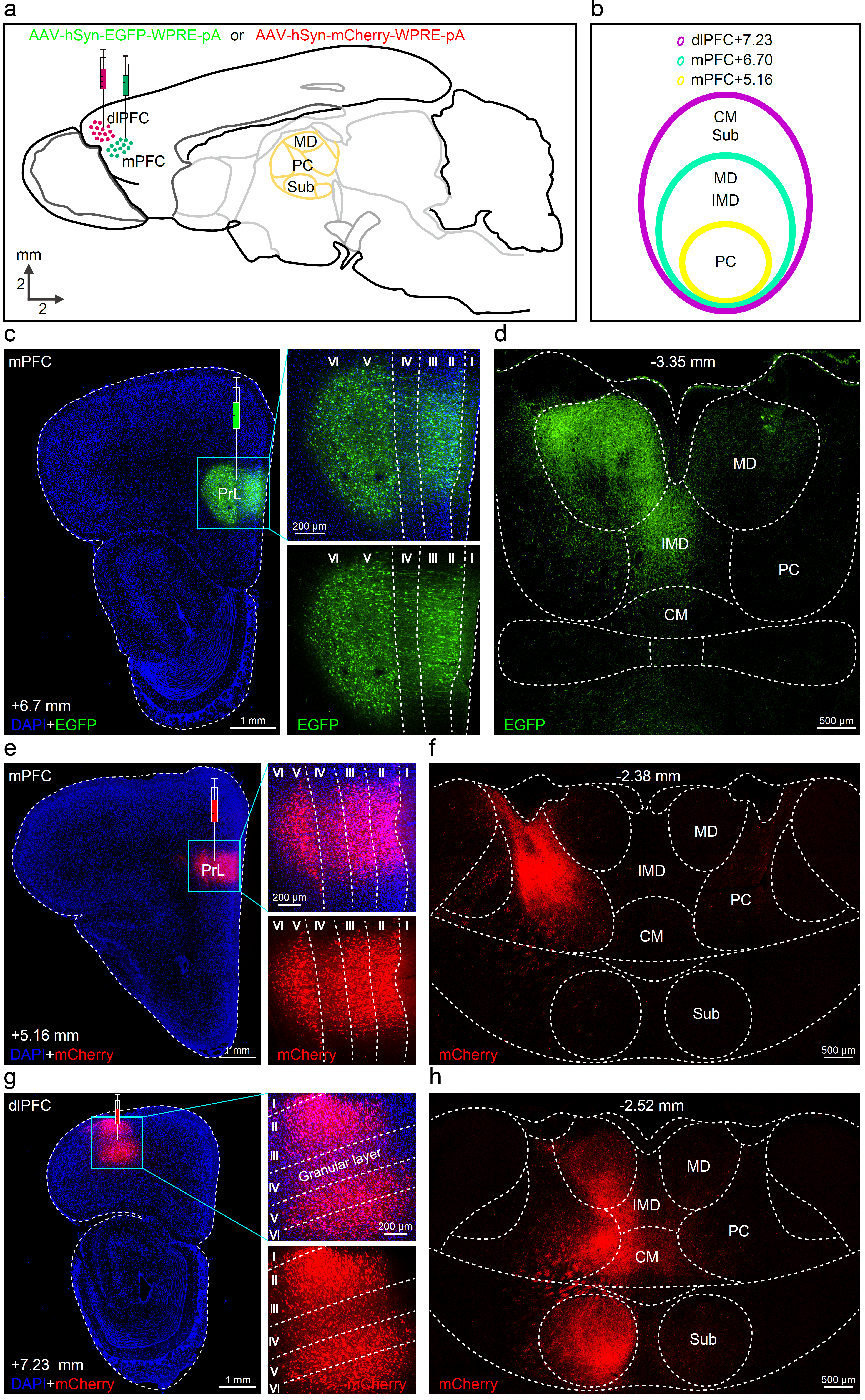

Supplement: Figure 3-1 — Comparison of the dlPFC and mPFC in descending projections to the thalamic nuclei. (a) AAV-hsyn-EGFP-WPRE-pA or AAV-hsyn-mCherry-WPRE-pA was injected into the dlPFC or mPFC, respectively. (b) The MD, IMD, and PC were the common regions receiving both the dlPFC and mPFC projections. (c-d) The EGFP-labelled neurons covered the L2-6 of the mPFC, in which the L4 looks like dsygranular. These neurons projected to the MD, PC, and IMD, with very few in the contralateral side of the MD. (e-f) The mCherry-labelled neurons were distributed also in the L2-6 of the mPFC, in which the L4 was much denser at this coordinate. These neurons sent projection terminals to the PC mainly. (g-h) The mCherry-labelled neurons were also distributed in the L2-6 of the dlPFC, where the L4 enriched with granular cells. These neurons projected to the MD, PC, IMD, CM, and Sub. PrL = prelimbic cortex; IMD = intermediodorsal nucleus; PC = paracentral nucleus; CM = central medial nucleus; Sub = submedius nucleus. Download Figure 3-1, TIF file. [file eneuro-11-ENEURO.0307-24.2024-s006.tif]
